# Modeling primary microcephaly with human brain organoids reveals fundamental roles of CIT kinase activity

**DOI:** 10.1172/JCI175435

**Published:** 2024-11-01

**Authors:** Gianmarco Pallavicini, Amanda Moccia, Giorgia Iegiani, Roberta Parolisi, Emily R. Peirent, Gaia Elena Berto, Martina Lorenzati, Rami Y. Tshuva, Alessia Ferraro, Fiorella Balzac, Emilia Turco, Shachi U. Salvi, Hedvig F. Myklebust, Sophia Wang, Julia Eisenberg, Maushmi Chitale, Navjit S. Girgla, Enrica Boda, Orly Reiner, Annalisa Buffo, Ferdinando Di Cunto, Stephanie L. Bielas

**Affiliations:** 1 Neuroscience Institute Cavalieri Ottolenghi, Turin, Italy.; 2 Department of Neuroscience “Rita Levi Montalcini,” University of Turin, Turin, Italy.; 3 Department of Human Genetics and; 4 Neuroscience Graduate Program, University of Michigan Medical School, Ann Arbor, Michigan, USA.; 5 Departments of Molecular Genetics and Molecular Neuroscience, Weizmann Institute of Science, Rehovot, Israel.; 6 Department of Molecular Biotechnology and Health Sciences, University of Turin, Turin, Italy.; 7 Department of Pediatrics, University of Michigan Medical School, Ann Arbor, Michigan, USA.

**Keywords:** Cell biology, Neuroscience, Genetic diseases, Neurodevelopment, Neuronal stem cells

## Abstract

Brain size and cellular heterogeneity are tightly regulated by species-specific proliferation and differentiation of multipotent neural progenitor cells (NPCs). Errors in this process are among the mechanisms of primary hereditary microcephaly (MCPH), a group of disorders characterized by reduced brain size and intellectual disability. Biallelic citron rho-interacting serine/threonine kinase (*CIT*) missense variants that disrupt kinase function (*CIT^KI/KI^*) and frameshift loss-of-function variants (*CIT^FS/FS^*) are the genetic basis for MCPH17; however, the function of CIT catalytic activity in brain development and NPC cytokinesis is unknown. Therefore, we created the *Cit^KI/KI^* mouse model and found that it did not phenocopy human microcephaly, unlike biallelic *Cit^FS/FS^* animals. Nevertheless, both *Cit* models exhibited binucleation, DNA damage, and apoptosis. To investigate human-specific mechanisms of *CIT* microcephaly, we generated *CIT^KI/KI^* and *CIT^FS/FS^* human forebrain organoids. We found that *CIT^KI/KI^* and *CIT^FS/FS^* organoids lost cytoarchitectural complexity, transitioning from pseudostratified to simple neuroepithelium. This change was associated with defects that disrupted the polarity of NPC cytokinesis, in addition to elevating apoptosis. Together, our results indicate that both CIT catalytic and scaffolding functions in NPC cytokinesis are critical for human corticogenesis. Species differences in corticogenesis and the dynamic 3D features of NPC mitosis underscore the utility of human forebrain organoid models for understanding human microcephaly.

## Abstract 2

New mammalian CIT models and organoid live-cell imaging reveal species-specific pathogenic mechanisms and highlight the sensitivity of developing brain to cytokinesis defects

## Graphical abstract

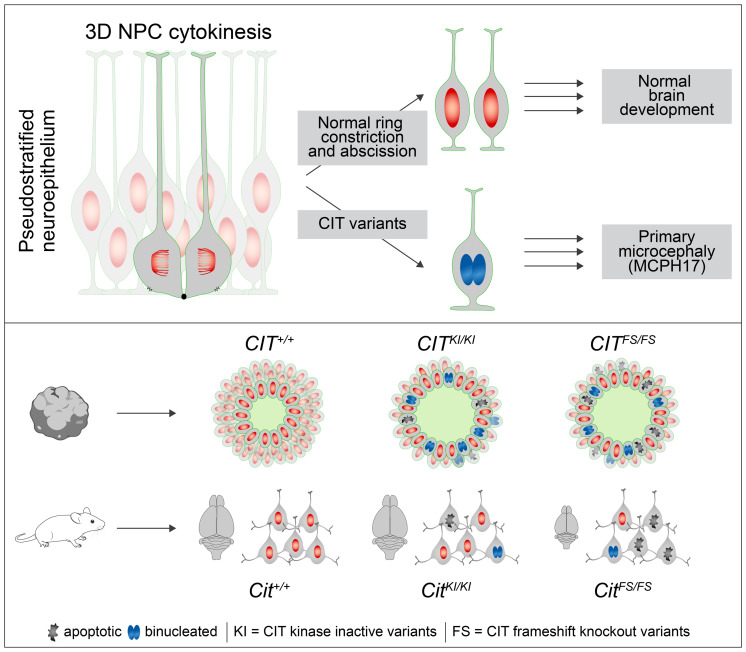

## Introduction

The mammalian cerebral cortex forms through a species-specific sequence of neural precursor cell proliferation and differentiation events (1, 2). Neuroepithelial stem cells expand through a series of symmetric proliferative divisions and then give rise to multipotent apical neural radial glia progenitor cells (aNPCs). In the ventricular zone, aNPCs divide symmetrically to produce additional multipotent progenitors and asymmetrically to produce basal neural radial glia progenitor cells (bNPCs) and intermediate progenitor cells, which continue to proliferate in the outer-subventricular zone or terminally differentiate into excitatory neuron subtypes (3). Expansion of multipotent aNPCs by symmetric proliferation and generation of progeny through asymmetric cell division are carefully regulated species-specific features of corticogenesis that contribute to the final size and folding of the cerebral cortex (1, 2, 4). Given the consequential role of aNPC symmetrical division in brain size, the apical-basal polarity of aNPCs is tightly controlled. aNPCs span the breadth of neuroepithelium with apical end-feet secured at the ventricular border by adhesion junctions that contain overlapping fate and polarity factors. The precise distribution of fate determinants between aNPC daughter progeny determines cell fate. During neurogenesis, cleavage furrow constriction of mitotic aNPCs in neuroepithelium is polarized; contractile ring constriction occurs from the basal-to-apical membrane to localize abscission at the apical border of the cell, adjacent to the ventricle (4). The position of the mitotic spindle relative to the apical border of aNPCs, shifting between perpendicular to oblique or even parallel, is also critical in the transition from symmetric to asymmetric divisions (5). The importance of this biology for corticogenesis is accentuated by primary hereditary microcephaly (MCPH) and microlissencephaly that result from errors in this developmental process (6, 7).

MCPH-associated proteins participate in many biological processes that affect aNPC proliferation and differentiation (7–11). Disentangling the complex physiological relationships between MCPH genes is complicated by the limited knowledge about their functional interactions and the difficulty of modeling human microcephaly in rodents, which do not recapitulate all details of human cortical development. Indeed, inactivation of MCPH genes in mice frequently produces significantly milder phenotypes than those observed in affected individuals (12, 13). We previously identified pathogenic biallelic variants in citron rho-interacting serine/threonine kinase (*CIT*) (OMIM ID: 605629) as the genetic basis for MCPH17 (14, 15). *CIT* represents a growing list of human genetic findings linking defects in cytokinesis machinery to clinical features of severe microcephaly. An overarching feature of these findings is their disproportionate effect on brain development despite widespread expression across tissues, highlighting a unique vulnerability of the brain to cytokinesis defects. *CIT* encodes a Ser/Thr protein kinase (CIT-K) that localizes to the contractile ring and midbody during cytokinesis. CIT-K is functionally critical for cytokinesis completion, midbody positioning, abscission, and maintenance of genomic integrity (16–20). CIT-K is characterized by an N-terminal kinase domain and C-terminal protein interaction domains. The C-terminal domains permit integration into the contractile ring and midbody, which is required for scaffolding functions. Biallelic *CIT* missense variants that disrupt kinase function (kinase-inactive [KI]) and loss-of-function (LOF) frameshift (FS) variants that destroy both kinase and scaffolding functions are the genetic basis of microcephaly and microlissencephaly, respectively (14, 15, 21, 22). The increasing severity of these clinical phenotypes indicate that both kinase and scaffolding functions are crucial for brain development.

In this report, we describe complementary mouse and human *CIT* KI models generated to discriminate the developmental mechanisms of CIT-K kinase function from the more severe *CIT* LOF corticogenesis phenotypes. *Cit* LOF rodent models displayed severe microcephaly as a result of altered neurogenesis, binucleation, and TP53-dependent apoptosis (18, 23, 24). *Cit^KI/KI^* mice do not phenocopy MCPH17 microcephaly, although they display aNPC molecular phenotypes consistent with *Cit* LOF models. Similar to the described mouse models, we observed progressively more severe molecular pathology between *CIT^KI/KI^* and *CIT*^FS/FS^ human 3D forebrain organoids. However, both genotypes showed striking alterations in the polarized organization and structural integrity of neural rosette neuroepithelium, revealing a function of CIT-K kinase activity in aNPC cytokinesis that is critical for early brain development. Our results underscore the relevance of species-specific sensitivity to CIT-K cytokinesis functions and further highlight human 3D forebrain organoids as a fundamental model to better understand human microcephaly.

## Results

### Brain size is unchanged in the Cit^KI/KI^ mouse model.

**Figure 1:**
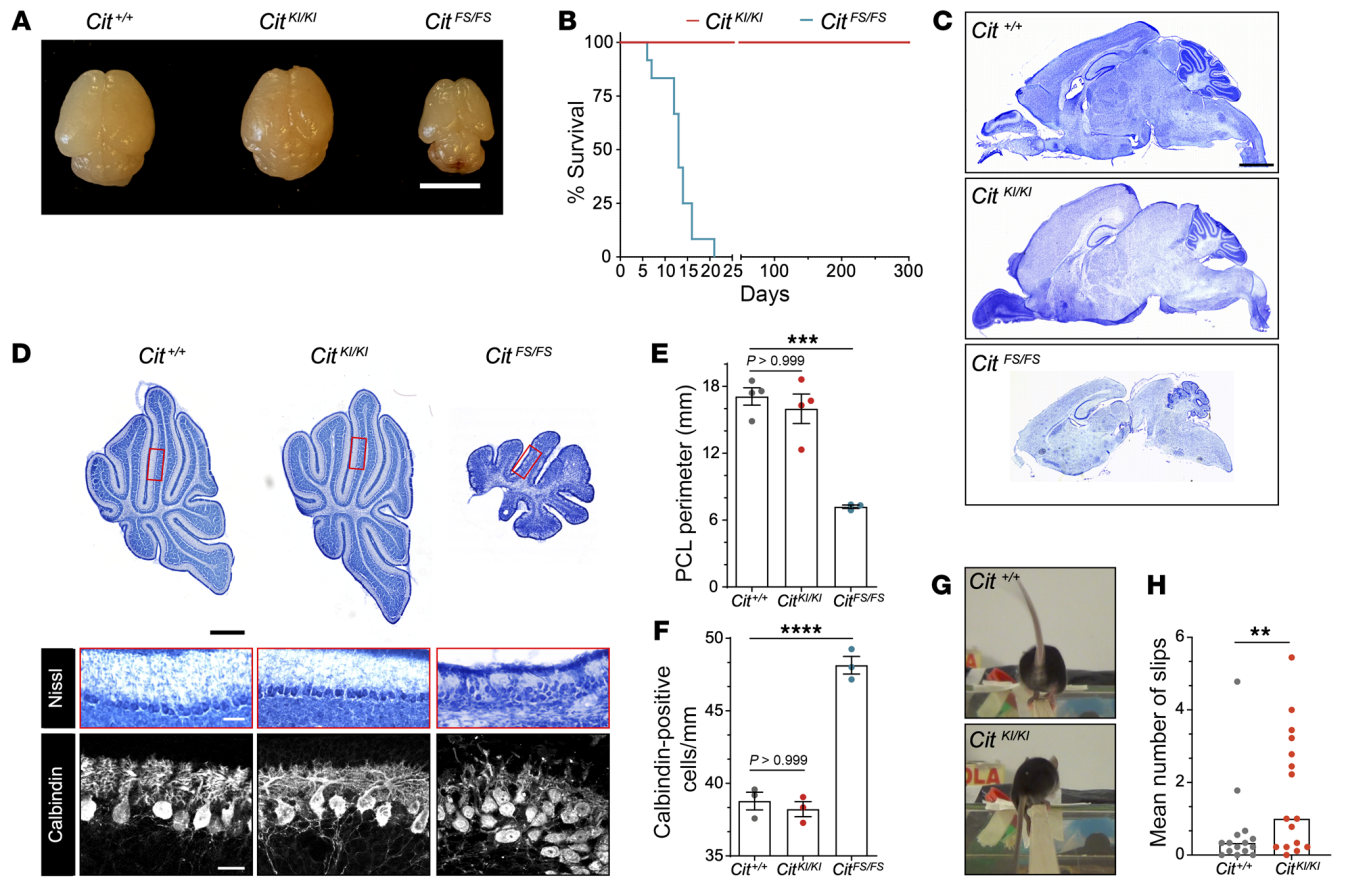
*Cit^KI/KI^* mice show grossly normal CNS morphological structure with an ataxic phenotype. (**A**) Representative image of *Cit^+/+^*, *Cit^KI/KI^*, and *Cit^FS/FS^* P10 brains. Scale bar: 5 mm. (**B**) Kaplan-Meier survival curves for *Cit^KI/KI^* mice versus *Cit^FS/FS^* (*n* = 12) mice. The log-rank (Mantel-Cox) test was used to compare survival between experimental groups (****P < 0.0001). (**C**) Cresyl Violet staining of sagittal sections of *Cit^+/+^*, *Cit^KI/KI^*, and *Cit^FS/FS^* P10 brains. Scale bar: 1 mm. (**D**) Upper panel: Cresyl violet staining of sagittal sections from the midline (vermis) of *Cit^+/+^*, *Cit^KI/KI^*, and *Cit^FS/FS^* P10 cerebella showing the anterior, central, and posterior sectors; scale bar: 500 μm. Middle panel: High-magnification Nissl stain of lobule V inset from the midline (vermis); scale bar: 50 μm. Lower panel: Immunofluorescence analysis for the Purkinje Cell marker calbindin 1 of sagittal sections of lobule V from the midline (vermis) obtained from P10 mice; scale bar: 25 μm. (**E**) Quantification of Purkinje cell layer (PCL) perimeter in the vermis of P10 *Cit^+/+^*, *Cit^KI/KI^*, and *Cit^FS/FS^* mice. (**F**) Quantification of Purkinje cell density per millimeter in the vermis of *Cit^+/+^*, *Cit^KI/KI^*, and *Cit^FS/FS^* P10 mice. Purkinje cells were stained for calbindin 1. (**G**) Representative picture extracted from videos of *Cit^+/+^* and *Cit^KI/KI^* mice slipping during beam walking test. (**H**) Quantification of the mean number of slips for 3 consecutive days of the beam-walking test on the same animal. Data indicate the mean ± SEM. **P < 0.01, ***P < 0.001, and ****P < 0.0001, by 1-way ANOVA followed by Bonferroni post hoc analysis (**E** and **F**) and unpaired, 2-tailed Student’s *t* test (**H**). Each dot represents an independent animal.

To investigate the role of CIT-K catalytic activity in brain development, we generated a new mutant mouse model. Two nucleotides were edited using traditional mouse embryonic stem cell–based (ESC-based) knockin technologies (25) to generate a *Cit* (c.376_377AA>GC; p.K126A) KI mouse line. This *Cit* missense substitution is similar to the *CIT* missense variants described in individuals with MCPH17 microcephaly (Supplemental Figure 1, A–E; supplemental material available online with this article; https://doi.org/10.1172/JCI175435DS1) (15). The K126A amino acid substitution was previously shown to fully abolish CIT-K kinase activity (Supplemental Figure 1B) (26). Homozygous *Cit^KI/KI^* pups were born at the expected Mendelian ratios. CIT-K had the highest expression in proliferating neural tissue. Western blot analysis of P4 cerebellum and cortex showed a 40% reduction in CIT-K expression in the cerebellum of *Cit^KI/KI^* mice and normal expression of CIT-N, a shorter brain-specific isoform lacking the amino terminal kinase domain (Supplemental Figure 1, F and G) (27, 28). We did not detect CIT-K expression in the cortex at this developmental time point (Supplemental Figure 1, F–I), consistent with limited postnatal aNPC proliferation (27–29). Since a 50% reduction of WT CIT-K has not been associated with a measurable phenotype (18, 23), comparing *Cit^KI/KI^* animals with homozygous frameshift (*Cit^FS/FS^*) and WT control mice allowed the phenotypic contribution of catalytic versus scaffolding activities of CIT-K to be assessed. Unlike *Cit^FS/FS^* mice, *Cit^KI/KI^* pups were indistinguishable from control littermates based on head morphology upon physical examination and were not microcephalic (Figure 1A and Supplemental Figure 2A). *Cit^KI/KI^* mice exhibited normal growth rates and were interfertile. We detected no perinatal lethality among *Cit^KI/KI^* pups, unlike *Cit^FS/FS^* mice, which die within 1 month of birth (Figure 1B). No significant alterations in cortical thickness, hippocampal structure, or cerebellar organization were detected in *Cit^KI/KI^* brains (Figure 1, C and D, and Supplemental Figure 2, B–E). The cerebellar cortex was the most affected brain region in *Cit^FS/FS^* mice (23), with a dramatic reduction of the granule cell layer, resulting in overall hypoplasia and increased linear density of Purkinje cells (Figure 1, D–F). While the absolute number of Purkinje cells was not reduced in *Cit^FS/FS^* cerebellum, they did display altered morphology, with immature dendritic arborizations. In contrast, *Cit^KI/KI^* cerebella were not morphologically distinguishable from controls throughout postnatal development (Figure 1, C–F, and Supplemental Figure 2, F–H). Likewise, *Cit^KI/KI^* mice did not exhibit seizure activity or the dramatic reeling phenotype that characterizes *Cit^FS/FS^* pathology (23). Behavioral characterization of WT and *Cit^KI/KI^* young mice at P8.5 did not show any differences in the maturation of ambulation, ataxic phenotype (i.e. spontaneous flipping on the back), or surface righting reflex that were evident in *Cit^FS/FS^* mice (Supplemental Figure 2, I–K). Conversely, *Cit^KI/KI^* mice exhibited poor performance on the negative geotaxis test of motor coordination compared with WT mice (Supplemental Figure 2L). This phenotype was still evident at 2 months of age, when the mice were assessed with the beam-walking test, consistent with a persistent motor defect (Figure 1, G and H, and Supplemental Videos 1 and 2). Overall, *Cit^KI/KI^* mice were normocephalic, which contrasted with the human microcephaly associated with similar biallelic missense variants. These data indicate that the hypomorphic *Cit*-KI allele preserved many essential CIT-K functions during brain development. Moreover, they indicate a differential requirement of kinase activity in human and mouse corticogenesis.

### Cellular phenotypes of apoptosis, DNA damage, and incomplete cytokinesis evident in Cit^KI/KI^ brain.

**Figure 2:**
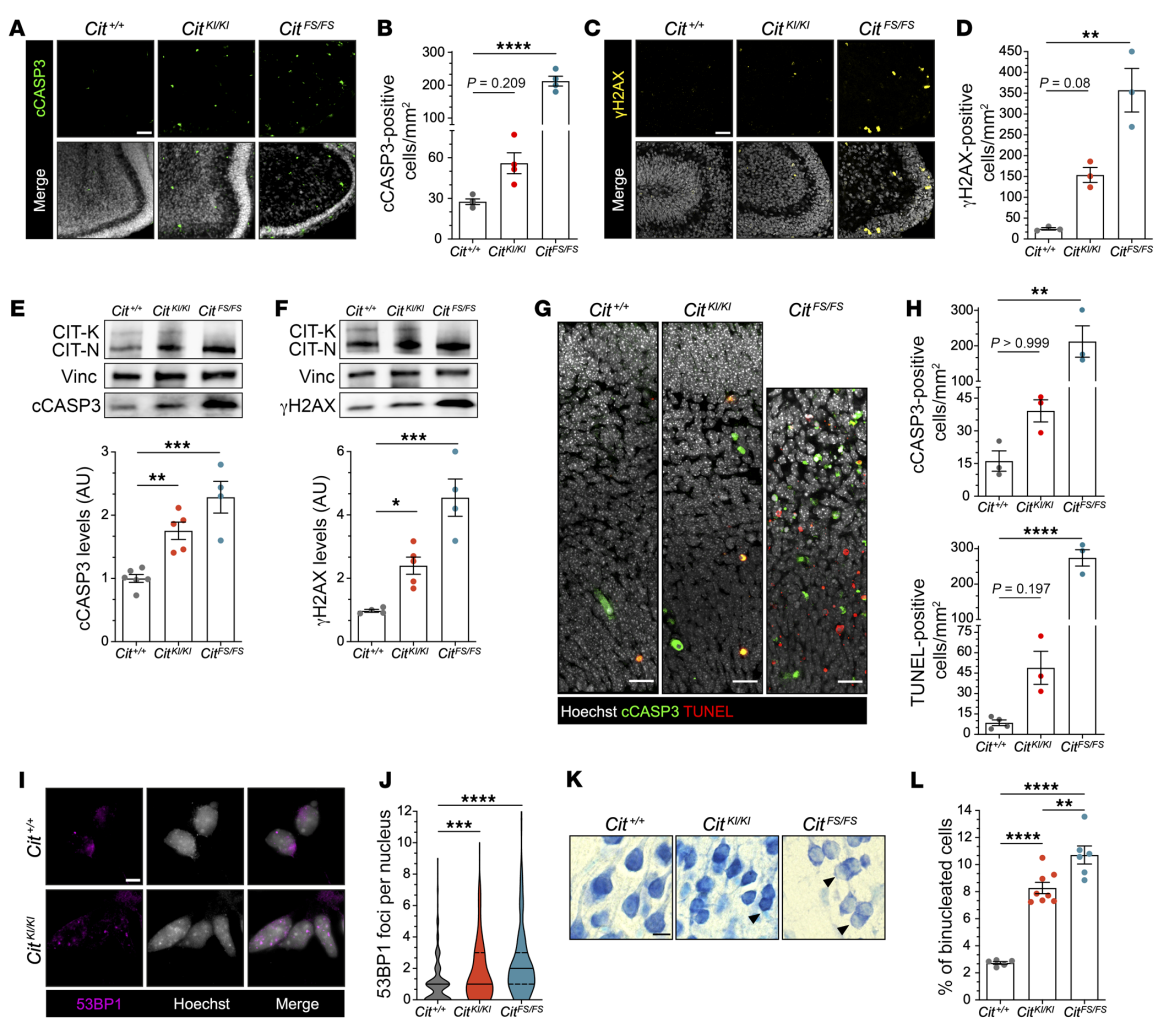
*Cit^KI/KI^* mice show increased apoptosis, DNA damage, and cytokinesis failure in CNS tissue, but less than *Cit^FS/FS^* mice. (**A**) Immunofluorescence analysis of P4 cerebella from mice of the indicated genotypes for the apoptotic marker cCASP3 (green) and Hoechst (gray). Scale bar: 25 μm. (**B**) Quantification of cCASP3^+^ cells in **A**. (**C**) Immunofluorescence analysis of P4 cerebella from mice of the indicated genotypes for the DNA damage marker γ*H2AX* (yellow) and Hoechst (gray). Scale bar: 25 μm. (**D**) Quantification of *γH2AX^+^* cells in **C**. (**E** and **F**) Western blot analysis of total lysate from P4 cerebella from mice of the indicated genotypes. The levels of cCASP3 (**E**) and *γH2AX* (**F**) were analyzed relative to the internal loading control vinculin (Vinc). Each dot represents an independent replicate. (**G**) Immunofluorescence analysis for the apoptotic marker cCASP3 (green), TUNEL assay (red), and Hoechst (gray) of E14.5 cortex obtained from embryos of the indicated genotypes. Scale bars: 10 μm. (**H**) Quantification of cCASP3 and TUNEL^+^ cells in (**G**). (**I**) Representative images of E12.5 NPCs from *Cit^+/+^* and *Cit ^KI/KI^* embryos, immunostained for 53BP1 (magenta) and Hoechst (gray) 18 hours after plating. Scale bar: 10 μm. (**J**) Quantification of 53BP1 nuclear foci in **I**; more than 250 cells were counted for each genotype in each replicate (*n* = 3). (**K**) High-power fields of Cresyl violet–stained coronal sections of P10 cortex obtained from mice of the indicated genotypes. Arrowheads indicate binucleated cells. Scale bar: 10 μm. (**L**) Quantification of binucleated cells in **K**. In microscopy quantifications, every dot represents an independent animal and at least 9 fields per genotype were analyzed. Data indicate the mean ± SEM. *P < 0.05, **P < 0.01,***P < 0.001, and ****P < 0.0001, by 1-way ANOVA followed by Holm-Šidák post hoc analysis.

*Cit^FS/FS^* neurons are characterized by binucleation, accumulation of DNA damage, and increased apoptosis, which are cellular phenotypes associated with cytokinesis failure (18, 23, 30). Previous studies have identified a prominent role of TP53-dependent apoptosis in the pathogenesis of microcephaly in this model (18). We therefore characterized these cellular phenotypes in the *Cit^KI/KI^* postnatal brain. Evaluation of *Cit^KI/KI^* P4 developing cerebellum revealed a slight increase in apoptotic cells, enriched in the inner portion of the external granule cell layer, as quantified by cleaved caspase 3 (cCASP3) immunofluorescence (Figure 2, A and B). This correlated with a significant increase in γH2AX staining in cerebellar cells, consistent with elevated DNA damage (Figure 2, C and D). While the frequencies of both apoptotic and γH2AX cells were increased in *Cit^KI/KI^* cerebellum relative to controls, the detected increases were higher in *Cit^FS/FS^* tissue. The increase in cCASP3 and γH2AX was confirmed by Western blotting, in which the highest level was again observed in *Cit^FS/FS^* samples (Figure 2, E and F). These *Cit^KI/KI^* phenotypes were still detected at P10, although apoptosis was not as severe as that observed at earlier time points (Supplemental Figure 3, A–D), suggesting that *Cit^KI/KI^* pathology was not sufficient to generate cerebellar hypoplasia, but implicated a developmental mechanism that correlated with the *Cit^KI/KI^* motor coordination deficits.

We also detected a modest increase in apoptosis and DNA double-stranded breaks in the developing *Cit^KI/KI^* neocortex (Figure 2, G and H). This correlated with an increase in 53BP1 foci per NPC nucleus, an established marker of DNA double-stranded breaks (Figure 2, I and J). Again, phenotypes in the *Cit^KI/KI^* samples were intermediate between the *Cit^FS/FS^* and control values. To estimate the *Cit* genotype–dependent effect on cytokinesis completion, we measured the ratio of binucleated neurons in Nissl-stained cortical tissue at the end of cortical development (P10). *Cit^KI/KI^* mice exhibited a significant increase in binucleation that was much closer to the levels detected in *Cit^FS/FS^* mice, unlike other phenotypes assessed (Figure 2, K and L). Throughout the cortex in *Cit^KI/KI^* mice at P10, we detected binucleated BLBP^+^ astrocytes and SOX10^+^ oligodendroglial cells scattered, which were observed at higher frequency in *Cit^FS/FS^* mice (Supplemental Figure 3, E and F), as previously demonstrated (30).

These results indicate that CIT kinase activity was required for completion of NPC cytokinesis, and incomplete cytokinesis correlated with a disproportional increase in binucleation in *Cit^KI/KI^* cortical tissue. However, the lower levels of DNA damage and apoptosis in *Cit^KI/KI^* tissue were not sufficient to produce the TP53-dependent severe microcephaly that characterizes *Cit^FS/FS^* mice.

### Human model of cortical development exhibits pathogenic mechanisms of CIT variants.

**Figure 3:**
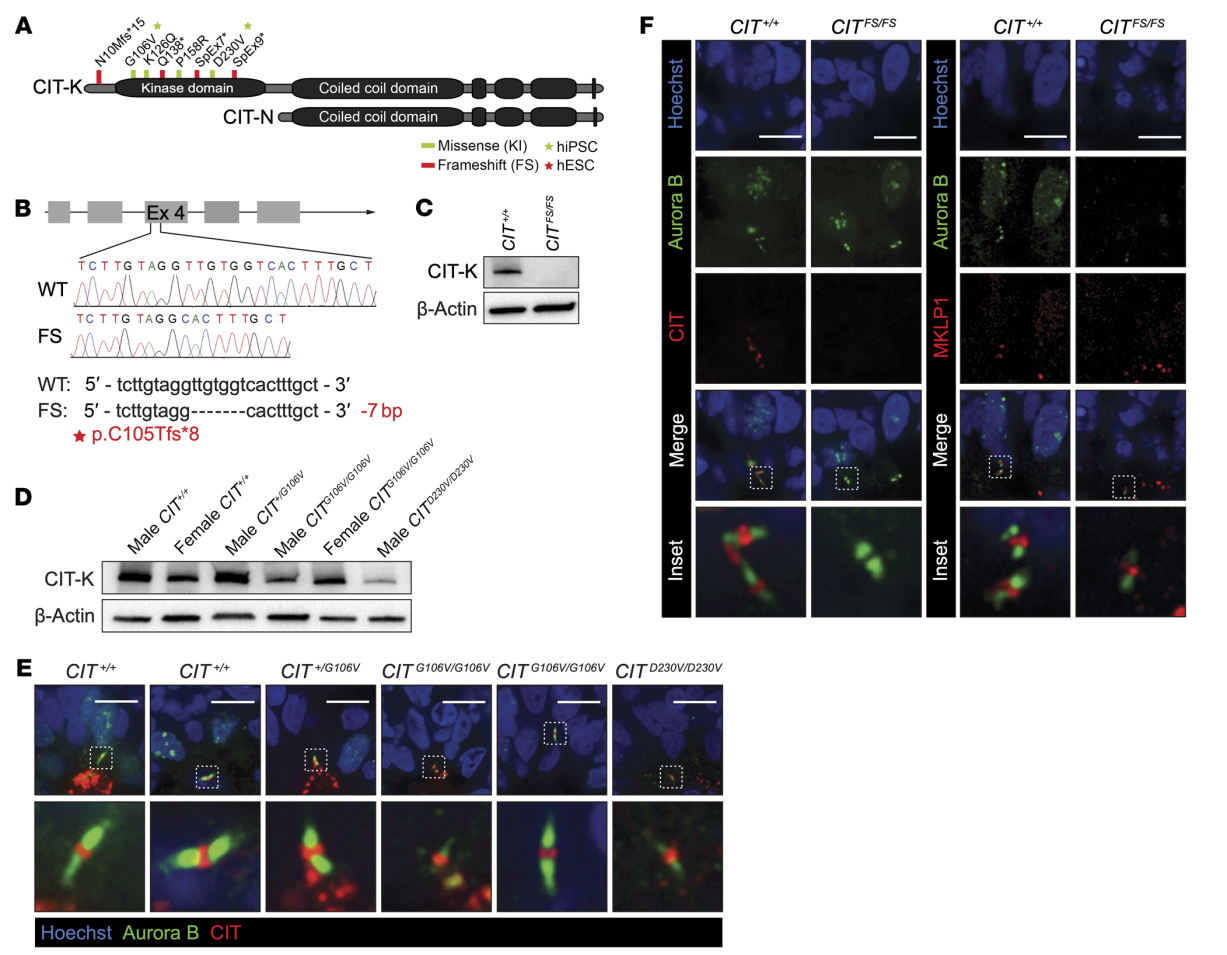
Modeling *CIT* variants using human models of neurodevelopment. (**A**) Scheme of CIT-K and CIT-N protein isoforms. Homozygous and compound heterozygous variants localized in the N-terminus and within the kinase domain of CIT-K. Missense variants are depicted in green, and LOF FS and splice (SpEx) variants are depicted in red. (**B**) CRISPR/Cas9-mediated targeting of the *CIT* locus at exon 4. Sequences and chromatograms of the 7 bp deletion are shown, with the corresponding alteration of the protein sequence highlighted in red. (**C**) Western blot analysis of CIT expression in NPCs of the indicated genotypes. The CIT-K isoform was absent in the CRISPR/Cas9-edited *CIT^FS/FS^* line. (**D**) Western blot analysis of CIT expression in NPCs of the indicated genotypes. Variability in CIT-K abundance was detected in *CIT^KI/KI^* NPCs compared with unaffected controls. (**E**) Immunofluorescence of Aurora B (green) midbody arms and CIT (red) show the presence of CIT in the midbody central bulge in the indicated genotypes of 35DD dorsal forebrain organoids. DNA stained with Hoechst (blue). Scale bars: 10 μm. (**F**) Immunofluorescence of Aurora B (green) midbody arms and the midbody central bulge markers CIT (red) and MKLP1 (red) in 35DD dorsal forebrain organoids. DNA stained with Hoechst (blue). CIT was absent in the midbody central bulge in *CIT^FS/FS^* 35DD organoids. Immunofluorescence with the midbody central bulge marker MKLP1 (red) shows the presence of this structure flanked by Aurora B (green) midbody arms in *CIT^FS/FS^* 35DD organoids. Scale bars: 10 μm.

The surprising finding that hypomorphic *Cit* missense variants that disrupt kinase activity were insufficient to produce microcephaly in mice led us to investigate how altered CIT-K catalytic and scaffolding functions contribute to microcephaly and microlissencephaly in humans. Toward this aim, we generated human in vitro models of dorsal forebrain development. Induced pluripotent stem cell (iPSC) lines from unaffected individuals (male and female *CIT^+/+^*), a related unaffected control (male *CIT^+/G106V^*), and affected individuals with *CIT* biallelic missense variants (female *CIT^G106V/G106V^* and male *CIT^G106V/G106V^*) were reprogrammed from primary fibroblasts (Figure 3A). iPSC lines from another affected male with MCPH17 (*CIT^D230V/D230V^*) allowed us to assess variability in the allelic series. To model *CIT* LOF, a clinically relevant homozygous deletion resulting in a FS and premature stop codon (NM_007174.3: c.312_318del; p.C105Tfs*8) was introduced in exon 4 of *CIT* by CRISPR/Cas9 genome editing of the female H9 human ESC (hESC) line to generate *CIT^FS/FS^* (Figure 3B). We evaluated the *CIT^KI/KI^* iPSC lines and edited *CIT^FS/FS^* hESC lines relative to *CIT^+/KI^* related-iPSC lines and a *CIT^+/+^* isogenic control line, respectively. The human pluripotent stem cell (hPSC) lines were analyzed by chromosomal microarray to rule out pathogenic copy number alterations (data not shown). hPSCs were differentiated into NPCs and dorsal forebrain organoids according to a dual-SMAD inhibition protocol (31). Neural rosettes in forebrain organoids recapitulate the evolutionarily conserved in vivo polarity of mammalian neuroepithelium and display polarized aNPC cytokinesis (32). The absence of the CIT-K isoform in the *CIT^FS/FS^* NPCs was confirmed by Western blotting (Figure 3C), and CIT-N is not expressed in this developmental cell type. As in the *Cit^KI/KI^* mouse model, we observed a variable reduction of CIT-K protein abundance in *CIT^KI/KI^* NPCs by Western blotting (Figure 3D). In organoids at 35 days of differentiation (35DD), the midbody formed at the apical border of aNPCs adjacent to the central lumen of neural rosettes (Figure 3E). This was comparable to polarized midbody placement adjacent to the lateral ventricle during in vivo corticogenesis (33). In control cells, CIT-K localized to the midbody central bulge, the structure formed between 2 dividing cells at the last stage of cytokinesis, and was flanked by Aurora B enrichment (Figure 3E). CIT-K harboring missense substitutions also correctly localized to the midbody central bulge in *CIT^KI/KI^* 35DD forebrain organoids (Figure 3E). In contrast, CIT-K was absent from the midbody central bulge in *CIT^FS/FS^* 35DD organoids. Counterstaining with mitotic kinesin-like protein 1 (MKLP1) highlighted the central bulge and confirmed that the gross midbody structure was preserved in the absence of CIT-K expression (Figure 3F).

### CIT^KI/KI^ and CIT^FS/FS^ organoids exhibit defects in pseudostratified neuroepithelium complexity.

**Figure 4:**
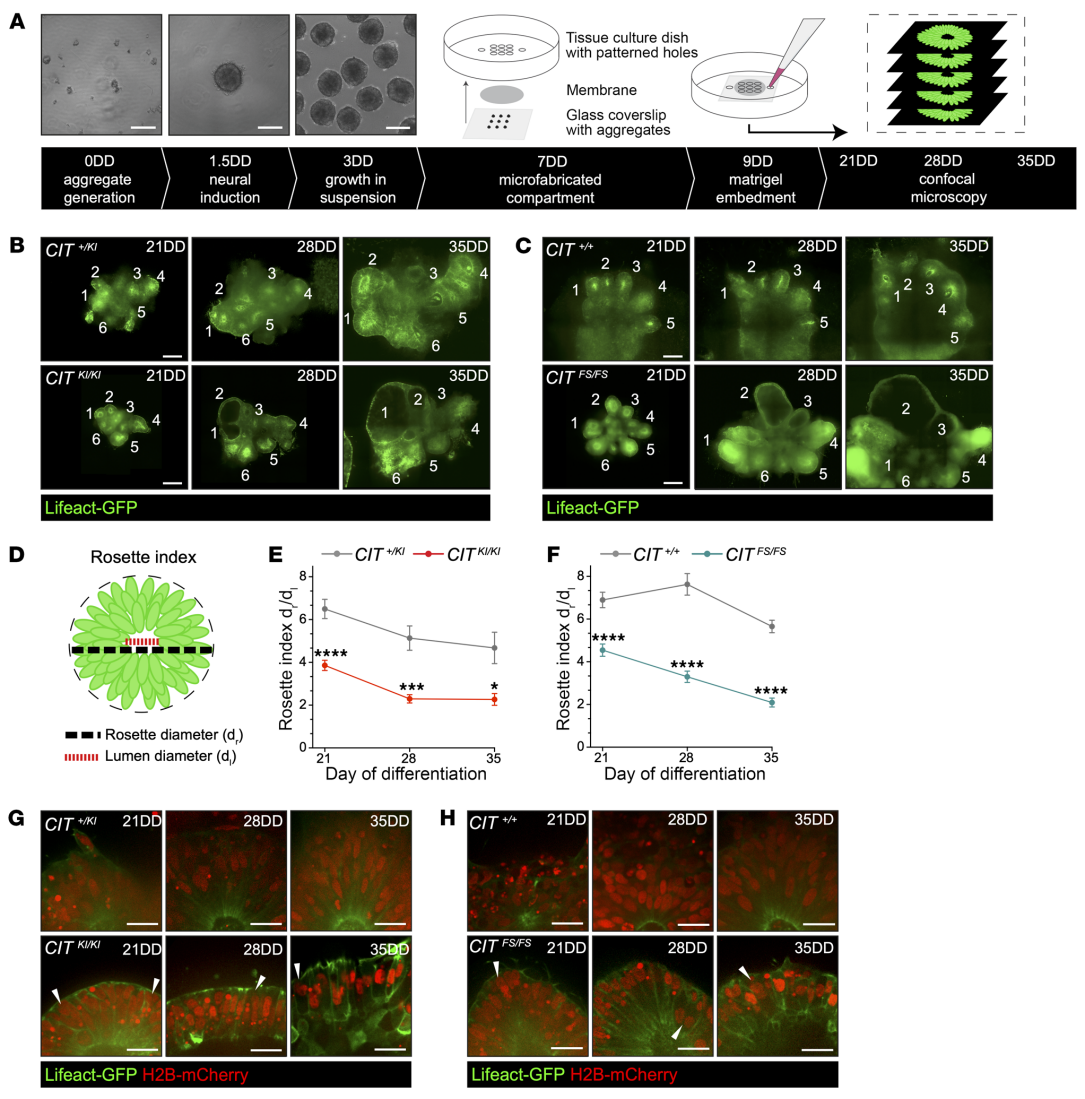
*CIT*-affected organoids demonstrate changes to pseudostratified neuroepithelium. (**A**) Schematic of dorsal forebrain organoid differentiation and microfabricated compartment generation with the corresponding time stamps. Neural differentiation begins with hPSC aggregate generation at 0DD, followed by neural induction at 1.5DD. An illustration of the microfabricated compartment depicts the orientation of compartment components. Neural aggregates are positioned on glass coverslips in a 3 × 3 pattern at 7DD, and the compartment is sealed with a UV adhesive. Neural differentiation medium fills the tissue culture dish to facilitate medium exchange to the compartment containing the developing tissue. Two days later, on 9DD, the compartment is embedded with Matrigel. Inverted confocal microscopy is performed on 21DD, 28DD, and 35DD. Scale bars: 200 μm. (**B** and **C**) Representative images of developing organoids and rosettes (white numbers) across time using Lifeact-GFP. Affected *CIT^FS/FS^* and *CIT^KI/KI^* rosettes exhibited large lumens and a reduction in neuroepithelial complexity. Scale bars: 250 μm. (**D**) Illustration of rosette measurements performed across *CIT* organoids, with diameter measurements and R_i_ calculations across organoid rosettes. (**E** and **F**) Quantification of R_i_ (d_r_/d_l_) in *CIT^+/KI^* and in *CIT^KI/KI^* organoids (**E**) and *CIT^+/+^* and in *CIT^FS/FS^* organoids (**F**). (**G** and **H**) Representative insets of developing rosettes across time using Lifeact-GFP and H2B-mCherry. Control rosettes maintained a pseudostratified neuroepithelium at all 3 time points, while many affected *CIT^KI/KI^* and *CIT^FS/FS^* rosettes showed transition toward a simple epithelial architecture. Multinucleated cells were apparent in the rosette, and examples are shown (white arrowheads). Scale bars: 25 μm. Quantification was done from a minimum of 2 independent compartment preparations per genotype. Each compartment contained 9 or fewer organoids per preparation. Data indicate the mean ± SEM. *P < 0.05, ***P < 0.001, and ****P < 0.0001, by repeated-measures, 2-way ANOVA.

To investigate *CIT* neuropathology in developing forebrain organoids, an allelic series of *CIT* hPSC lines stably expressing fluorescent markers to label nuclei (H2B-mCherry) and actin (Lifeact-GFP) were created using the PiggyBac transposase method (34). Labeled hPSCs were dissociated into single cell suspensions at 0DD to generate aggregates for dorsal forebrain organoid neural differentiation (Figure 4A). Aggregates were enclosed in a microfabricated compartment of fixed height between a polycarbonate membrane and glass coverslip at 7DD using an established protocol (35). The polycarbonate membrane facilitated medium exchange, while the glass coverslip permitted inverted optical imaging. The compartments were filled with Matrigel hydrogel at 9DD to mimic an in vivo environment and stabilize organoid positioning. Confocal microscopy of live organoids in culture was performed at 21DD, 28DD, and 35DD to investigate the effects of biallelic *CIT* KI and FS variants on organoid development. Reimaging of the same neural rosettes at progressively later days of neural differentiation in culture was critical for revealing *CIT^KI/KI^* and *CIT^FS/FS^* defects. All hPSCs carrying biallelic *CIT* missense variants (female *CIT^G106V/G106V^*, male *CIT^G106V/G106V^*, and male *CIT^D230V/D230V^*) were labeled and differentiated into forebrain organoids. Data for these genotypes were pooled for analysis and are shown as *CIT^KI/KI^*. Immunofluorescence assessment of SOX2-labeled (NPCs), DCX-labeled (immature neurons), and BCL11B-labeled (cortical neurons) cells in *CIT^+/+^*, *CIT^+/KI^*, *CIT^KI/KI^*, and *CIT^FS/FS^* 35DD fixed organoids revealed tissue differentiating along a dorsal forebrain fate (Supplemental Figure 4, A and B). Consistent with *CIT* pathology and in line with previous publications, we detected binucleated BCL11B cells in *CIT^KI/KI^* and *CIT^FS/FS^* organoids (Supplemental Figure 4, C and D), indicating that *CIT* variants did not preclude the differentiation of mature cortical neuron subtypes.

Cytoarchitecture of 21DD organoids consisted of neural rosettes composed of aNPCs extending from the apical (inner) border of a central lumen to a basal (outer) border. Interphase aNPC nuclei were densely packed, creating a pseudostratified neuroepithelium. *CIT^KI/KI^* and *CIT^FS/FS^* neural rosette cytoarchitecture was comparable to that of controls at 21DD, though a small dilation of the central lumen was detectable (Supplemental Figure 4, E and F, and Figure 4, B and C). We observed striking changes in the neuroepithelial organization of the same neural rosettes for *CIT*-affected genotypes reimaged at 28DD and 35DD (Figure 4, B and C). While control heterozygous *CIT^+/KI^* and *CIT^+/+^* rosettes maintained pseudostratification at 28DD and 35DD, multiple *CIT^KI/KI^* and *CIT^FS/FS^* neural rosettes transitioned from pseudostratified tissue to a simple epithelial cytoarchitecture with less cellular complexity.

Reduced *CIT^KI/KI^* and *CIT^FS/FS^* tissue complexity was directly correlated to increased diameter of the neural rosette and the central lumen. We developed the rosette index (R_i_) to assess this phenotype. Rosette index (R_i_ = d_r_/d_l_) is a ratio of neural rosette outer diameter (d_r_) relative to the inner lumen diameter (d_l_), which is proportional to the complexity of neural rosette pseudostratification (Figure 4D). The R_i_ of *CIT^+/KI^* (R_i_= 6.49) and *CIT^+/+^* (R_i_ = 6.89) control organoids was comparable at 21DD and remained relatively stable between 21DD and 35DD (Figure 4, E and F). In contrast, *CIT^KI/KI^* and *CIT^FS/FS^* neural rosettes exhibited a significantly lower R_i_ relative to that of their respective controls at 21DD and continued to decrease when measured again at 28DD, with *CIT^KI/KI^* at R_i_ = 2.29 and *CIT^FS/FS^* at R_i_ = 3.29. Between 28DD and 35DD, the R_i_ of *CIT^FS/FS^* rosettes continued to decline (R_i_ = 2.09), while the R_i_ of *CIT^KI/KI^* organoids plateau, despite remaining significantly lower than the *CIT^+/KI^* control R_i_ (Figure 4, E and F). While the absolute *CIT^KI/KI^* and *CIT^FS/FS^* R_i_ values do not scale exactly with severity in affected individuals, the phenotypic trend in both allelic series is comparable and the difference is highly significant, demonstrating the reproducibility of this phenotype. Of note, cells in these structures were clearly multinucleated with pyknotic nuclei, implicating cytokinesis defects and apoptosis in the pathogenesis of this phenotype (Figure 4, G and H). The comparable cytoarchitectural defects observed in *CIT^KI/KI^* and *CIT^FS/FS^* neural rosettes indicate that CIT-K kinase activity is critical for early human corticogenesis.

### CIT-K is critical for cytokinesis in 3D tissue.

**Figure 5:**
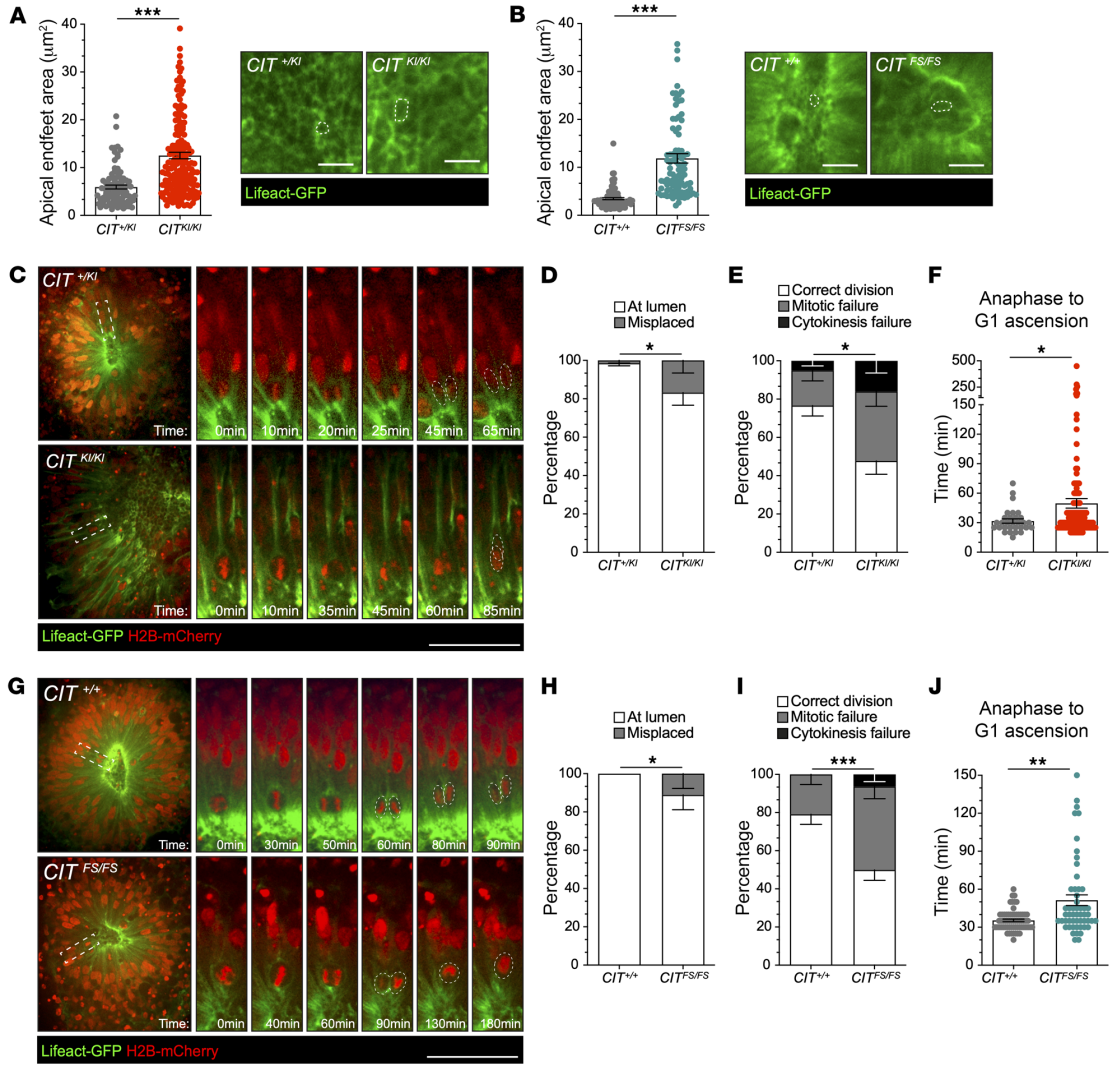
*CIT*-affected organoids show aNPC apical endfeet surface area increases and mitotic defects. (**A** and **B**) Apical endfeet surface area and representative images of 35DD organoid apical surface labeled with Lifeact-GFP in microfabricated compartments. The apical endfeet surface area was increased in affected *CIT^KI/KI^* (**A**) and *CIT^FS/FS^* (**B**) organoids compared with *CIT^+/KI^ and CIT^+/+^* control organoids. Scale bars: 10 μm. (**C**) Representative images from videos of *CIT^+/KI^* and *CIT^KI/KI^* rosettes with aNPC mitotic division (white dashed rectangles) at the apical surface. Panel shows the time details of this division. Scale bar: 50 μm. (**D**) Quantification of the percentage of division occurring at the central lumen or misplaced from the lumen in **C**. (**E**) Quantification of the percentage of correct division, mitotic failure, and cytokinesis failure in **C**. (**F**) Measurement of the time spent from anaphase to G_1_ ascension in *CIT^+/KI^* and *CIT^KI/KI^* aNPC divisions in 35DD organoid rosettes. Each dot indicates a single dividing cell. (**G**) Representative images from videos of *CIT^+/+^* and *CIT^FS/FS^* rosettes with aNPC mitotic division (white dashed rectangles) at the apical surface. Panel shows the time details of this division. Scale bar: 50 μm. (**H**) Quantification of the percentage of division occurring at the central lumen or misplaced from the lumen in **G**. (**I**) Quantification of the percentage of correct division, mitotic failure, and cytokinesis failure in **G**. (**J**) Measurement of the time spent from anaphase to G_1_ ascension in *CIT^+/+^* and *CIT^FS/FS^* aNPC divisions in 35DD organoid rosettes. Each dot indicates a single dividing cell. Quantification was done from a minimum of 2 independent compartment preparations per genotype. The compartments each contained 9 or fewer organoids per preparation. Data indicate the mean ± SEM. *P < 0.05, **P < 0.01, and ***P < 0.001, by unpaired, 2-tailed Student’s *t* test for the apical endfeet area (**A** and **B**), Fisher’s exact probability test for the percentage distribution (**D**, **E**, **H**, and **I**), and Mann-Whitney *U* test for the time from anaphase to G_1_ ascension (**F** and **J**).

Growing evidence indicates that disruption of polarized cytokinesis in 3D epithelial tissue can disrupt the coordination of abscission and formation of adhesion junctions between daughter and neighboring cells (36, 37). Likewise, incomplete cytokinesis would fail to divide the apical membrane that attaches aNPCs to the central lumen of neural rosettes. These features were evaluated to determine their contribution to the cytoarchitectural changes observed in *CIT*-affected organoids. Confocal imaging of rosette lumens in Lifeact-GFP–labeled 35DD organoids allowed the surface area of the aNPC apical attachments, also described as apical endfeet, to be quantified (Supplemental Figure 5A). The GFP outline of apical endfeet were manually traced, and the surface area was quantified (Supplemental Figure 5A). The average area of *CIT^+/KI^* apical endfeet at 35DD was 5.9 μm^2^, whereas the *CIT^KI/KI^* apical endfeet area was significantly increased to 12.5 μm^2^ (Figure 5A). We also observed an increase of *CIT^FS/FS^* apical endfeet that measured 11.9 μm^2^ compared with control *CIT^+/+^* apical endfeet, which averaged 3.4 μm^2^ (Figure 5B). Similar results were obtained by immunofluorescence analysis of fixed 35DD organoids, in which N-cadherin (CDH2) was used to determine the outline of apical endfeet surface area (Supplemental Figure 5, B–D).

CIT-K is an attractive candidate to coordinate apical placement of aNPC abscission. Failure to coordinate polarized cytokinesis with adhesion junction formation can result in loss of apical attachments and delamination, a potential mechanism that may contribute to the increased size of aNPC apical endfeet and changes to tissue complexity. We assessed cell division placement in live-cell imaging data and by immunohistochemistry of fixed organoid tissue. According to both analyses, a high percentage of aNPC cell divisions were misplaced relative to the central lumen in *CIT^KI/KI^* organoids; approximately 17% of mitotic cells were not located at the central lumen, as is expected during normal aNPC symmetrical expansion (Figure 5, C and D) (38). This finding was corroborated by immunostaining of midbody and apical localized markers in fixed 35DD organoids (Supplemental Figure 6, A and B). An increased percentage of misplaced Aurora B–marked midbodies, displaced relative to the N-cadherin apical surface–marked central lumen, was quantified in *CIT^KI/KI^* tissue compared with control organoid tissue (Supplemental Figure 6, A and B). An increase of misplaced mitotically active aNPCs was also present in *CIT^FS/FS^* organoids, as shown by live imaging (Figure 5, G and H) and analysis of midbody position with Aurora B staining of fixed organoid tissue (Supplemental Figure 6, C and D). Increased apical endfeet surface area and frequency of ab-luminal midbody placement in *CIT^KI/KI^* and *CIT^FS/FS^* neural rosettes correlated with the simplification of cytoarchitecture in the corresponding organoids and highlighted pathogenic mechanisms that implicate a vulnerability of the developing brain to aNPC cytokinesis defects. Additional analysis is required to determine if this phenotype correlates to the enlarged ventricles revealed by histological evaluation of *CIT^FS/FS^* postmortem brain tissue (14).

### CIT^KI/KI^ and CIT^FS/FS^ organoids exhibit mitotic defects with genotype-dependent severity.

Cytokinesis delay and increased cell binucleation due to cytokinesis failure are a conserved cell-autonomous pathology observed in *Cit*-KO rodent models and 2D NPCs derived from *CIT*-affected hPSC lines (14, 23, 39). To evaluate this dynamic and transient cell biology in 3D aNPC mitosis, we performed 2-channel, live-cell imaging of H2B-mCherry/Lifeact-GFP–labeled 35DD organoids at 5- to 10-minute intervals over 8–16 hours (Figure 5, C and G). A markedly higher frequency of profound mitotic defects, such as aNPC mitotic failure (stalled cells that do not complete division after prometaphase), cytokinesis failure (cells with 2 decondensed nuclei ascending in a single Lifeact-GFP–encircled NPC soma after furrowing), and an altered cleavage plane angle (Supplemental Figure 6, E–H) were observed in both *CIT^KI/KI^* and *CIT^FS/FS^* organoids (Supplemental Videos 3–6). Specifically, mitotic failure occurred in 18% of *CIT^+/KI^* aNPCs and doubled to 36% in *CIT^KI/KI^* aNPCs (Figure 5E). Likewise, mitotic failure occurred in 23% of *CIT^+/+^* aNPCs and 44% in *CIT^FS/FS^* aNPCs (Figure 5I). An increase in failed cytokinesis was detected in both affected genotypes (Figure 5, E and I). Frequent binucleated cells observed undergoing multipolar mitosis is further evidence of this cellular pathology. Conversely, *CIT*-genotype had a mild effect on the timing of aNPC mitosis for aNPCs that completed mitosis and continued their interkinetic nuclear migration. *CIT^KI/KI^* organoids did not exhibit a significant increase in aNPC mitotic duration. However, we observed a significant delay in aNPC progression from anaphase to nuclei ascension in *CIT^KI/KI^* organoids. On average, *CIT^KI/KI^* aNPCs took approximately 50 minutes compared with approximately 30 minutes for *CIT^+/KI^* control aNPCs to progress from anaphase to nuclei ascention (Figure 5F). In *CIT^FS/FS^* organoids, the delay in aNPC progression from anaphase to nuclei ascension increased to approximately 52 minutes compared with approximately 36 minutes for *CIT^+/+^* control aNPCs (Figure 5J). Together, these data demonstrate that CIT-K kinase activity was necessary for mitotic progression and timely abscission in aNPCs.

### Increased DNA damage and apoptosis are a conserved CIT neuropathology.

**Figure 6:**
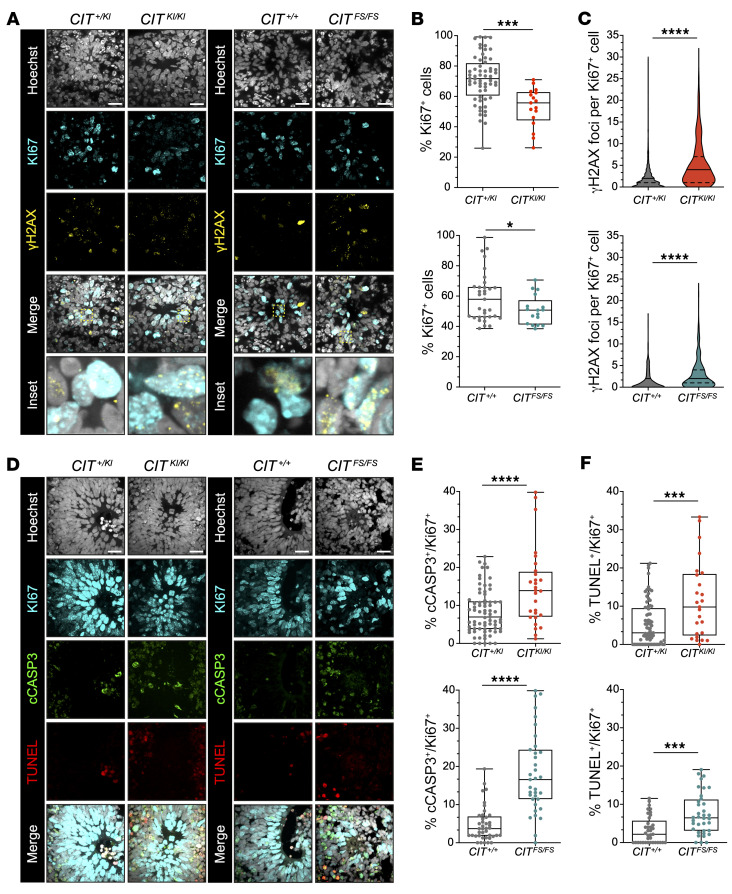
*CIT*-affected organoids show accumulation of DNA damage and apoptosis. (**A**) Representative images of rosettes for the indicated *CIT* genotypes for the DNA damage marker γH2AX (yellow), the proliferation marker Ki67 (cyan), and Hoechst (gray). Scale bars: 20 μm. (**B** and **C**) Quantification of Ki67^+^ cells (**B**) and γH2AX foci per Ki67^+^ cell (**C**). More than 500 cells were counted for each genotype in each replicate. (**D**) Representative images of rosettes for the indicated *CIT* genotypes for the apoptotic marker cCASP3 (green), TUNEL (red), the proliferation marker Ki67 (cyan), and Hoechst (gray). Scale bars: 20 μm. (**E** and **F**) Quantification of cCASP3 (**E**) and TUNEL (**F**) staining per Ki67^+^ cells. Every dot represents a neural rosette. Quantification was done from a minimum of 3 independent organoid preparations. Data indicate the mean ± SEM. *P < 0.05, ***P < 0.001, and ****P < 0.0001, by unpaired, 2-tailed Student’s *t* test (**B**, **E**, and **F**) and Mann-Whitney *U* test (for γH2AX foci in **C**).

DNA damage and apoptosis are a cellular pathology that can have measurable negative effects on aNPC proliferation, R_i_, and cellularity required for pseudostratification. We analyzed these features by immunofluorescence of fixed organoid tissue differentiated from fluorescent reporter–free hPSC lines. Ki67, a marker of proliferation, showed a significant reduction in 35DD *CIT^KI/KI^* and *CIT^FS/FS^* organoids (Figure 6, A and B). Unlike other cellular phenotypes, this reduction was higher in *CIT^KI/KI^* organoids than in *CIT^FS/FS^* organoids. DNA damage and apoptosis are established mechanisms for reducing proliferating cells from the neural progenitor pool and were evaluated to better understand the Ki67 reduction across genotypes. A robust increase in γH2AX foci in Ki67^+^ *CIT^KI/KI^* and *CIT^FS/FS^* NPCs revealed an accumulation of DNA damage in affected tissue (Figure 6C). This is consistent with previous work showing that *CIT* LOF variants generates DNA damage accumulation in developing mouse brains and postmortem tissue of an affected individual (23, 30, 40) and adds the observation that loss of CIT-K kinase function is sufficient to generate DNA damage. Induction of apoptosis was associated with incomplete cytokinesis in mice (23), human brain tumor lines (40, 41), in vitro NPCs (15), and affected individuals (14). Proliferating Ki67^+^ cells in both *CIT^KI/KI^* and *CIT^FS/FS^* organoids exhibited a significant increase in cells colabeled with either cCASP3 or TUNEL relative to controls (Figure 6, D–F). Of note, TUNEL^+^ cells were more pronounced in the *CIT^KI/KI^* Ki67 progenitors, whereas cCASP3^+^ cells were more pronounced in *CIT^FS/FS^* tissue (Figure 6, D–F). In this comparison, the TUNEL assay may detect DNA damage and underestimate apoptosis (42). Taken together, these results suggest that damaged DNA may have accumulated in *CIT^KI/KI^* NPCs more frequently, resulting in a growth-arrested state in *CIT^KI/KI^* rosettes, while NPCs progressed more frequently to apoptosis in *CIT^FS/FS^* rosettes.

## Discussion

In this study, we differentiated between the role of hypomorphic CIT-K signaling from *CIT* LOF defects in human and mouse brain development. The high frequency of binucleated neurons in mouse and human biallelic missense kinase inactive models indicates that the role of CIT-K in completion of cytokinesis was conserved between mouse and human models. However, this hypomorphic cytokinesis defect did not generate significant alterations in size, gross structure, or cytoarchitecture in *Cit^KI/KI^* mice, as has been described for *Cit^FS/FS^* mice. These differences between the hypomorphic and LOF *Cit* mouse models underscore the shared human *CIT^KI/KI^* and *CIT^FS/FS^* phenotype of simplified neuroepithelium in neural rosettes, suggesting a human-specific role for the catalytic activity of CIT-K in spatially organized cytokinesis, which is crucial for maintaining aNPC mitotic symmetry. The increased apoptosis in LOF neural tissues of both species highlights DNA damage–dependent apoptosis as a conserved determinant of brain size, which may explain the more severe microcephaly in *CIT* LOF models and affected individuals with these variant types. The use of 3D brain organoids underscored a new role of CIT-K and its kinase-dependent developmental dynamics in polarized, pseudostratified cytoarchitecture that was not possible to study using 2D in vitro cultures. Together, these considerations further highlight human forebrain organoids derived from cells of affected individuals as a fundamental resource to study the effect of MCPH-associated pathogenic genetic variants on neurodevelopment.

Rodent models have been critical for uncovering the fundamental biology of corticogenesis and pathogenic mechanisms contributing to primary microcephaly (43). The *Flathead* rat and the *Cit*-KO mouse phenocopy the severe microcephaly caused by human biallelic *CIT^FS/FS^* variants (23, 43). Affected animals have reduced brain size and cellularity owing to failed cytokinesis, binucleated neural tissue, DNA damage, and apoptosis. Reduced brain size is preferentially attributed to elevated apoptosis, as inactivation of TP53 during *Cit^FS/FS^* brain development rescues this phenotype and results in a corresponding increase in binucleated neurons (18). Our data indicate that this is a conserved pathogenic mechanism, with a prolonged late M-phase, binucleation, and apoptosis observed in human *CIT^FS/FS^* organoids, corroborating in vivo data and demonstrating the contribution of apoptosis to the pathogenic mechanism of microcephaly disorders (44). Although we did not observe microcephaly in the *Cit^KI/KI^* model, we show that there was a shared molecular pathology with other *CIT* models. Evidence of DNA damage accumulation and apoptosis was present, but to a lesser extent than in *Cit^FS/FS^* mice, suggesting that there is a threshold that can be tolerated before apoptotic pathways profoundly affect brain size. In human brain organoids, altered mitotic dynamics and elevated apoptosis were detected in *CIT^KI/KI^* organoids but, again, to a lesser extent than in *CIT^FS/FS^* organoids. It is unclear whether human brain development is more sensitive to the molecular consequences of hypomorphic CIT-K kinase activity, or if the increased duration of human gestation contributes to the *CIT^KI/KI^* microcephaly phenotype of affected individuals. Meanwhile, both human and mouse models indicate that CIT-K kinase functions are not fully redundant with scaffolding functions during cytokinesis.

The discovery that hypomorphic *CIT* alleles are the basis of primary microcephaly highlighted a previously unappreciated role for CIT-K kinase activity as a determinant of brain size (15). Human brain organoid models provided insight into the role of CIT-K kinase activity in corticogenesis and demonstrated the vulnerability of the developing brain to cytokinesis defects. Reimaging of the same GFP-labeled *CIT^KI/KI^* and *CIT^FS/FS^* neural rosettes revealed a similar genotype-driven effect on cytoarchitecture integrity. Normally, polarized aNPCs attach to a small central lumen to form pseudostratified neural rosettes. In *CIT*-affected organoids, multiple neural rosettes progressively transformed from a pseudostratified cytoarchitecture at 21DD to large lumens surrounded by a layer of cells resembling simple epithelial tissue at 28DD and 35DD. Similar cytoarchitecture defects in *CIT^KI/KI^* and *CIT^FS/FS^* neural rosettes indicate that CIT-K kinase activity was required to maintain the neuroepithelial polarity important for cortical morphogenesis as the cellular complexity of human neuroepithelium increases during development. The role of polarized cytokinesis of 3D aNPCs in neuroepithelium may explain the unique vulnerability of the developing brain to kinase disruption and implicates CIT-K kinase activity in coordinating this cell biology.

Polarized cytokinesis allows aNPCs to perpetuate their polarity (45, 46). Abscission at the apical border ensures precise distribution of fate determinants between daughter progeny. Moreover, abscission that occurs apically to newly formed cell-cell adhesion junctions between daughter and neighboring cells maintains the attachment of progeny to the ventricular membrane/central lumen (45, 47). Last, apical endfeet, the aNPC membrane section that attaches to the central lumen, are divided to ensure that each pseudostratified aNPC progeny has an apical contact as aNPCs divide symmetrically (45, 47). *CIT* organoids analyzed by live imaging and immunofluorescence showed increased frequency of misplaced mitotic events relative to the apical border of the rosette central lumen and larger apical endfeet size in *CIT^KI/KI^* and *CIT^FS/FS^* neural rosettes, providing evidence that the fidelity of polarized cytokinesis is disrupted in *CIT*-affected aNPCs. Alternatively, reduced cytoarchitectural complexity of *CIT*-affected neural rosettes could occur through premature differentiation due to altered fate determinant allocation and delamination of aNPCs from the central lumen that do not form adhesion junctions in 3D tissue. These changes, affecting the number of cells connected to the apical surface, could also influence the size of the remaining apical endfeet. This pathology highlights an important role for polarized cytokinesis in the cytoarchitectural complexity of pseudostratified neural rosettes.

Elegant studies in the *Drosophila* pupa epithelium demonstrated that the coordination of cleavage furrow constriction of daughter cells requires neighbor membrane deformation and a focal disengagement of their E-cadherin adhesive contact (36). Likewise, daughter cells are ultimately integrated into epithelial tissue when midbody formation occurs in a polarized configuration relative to adhesion junction assembly. While this work exemplifies the role of cellular neighbors in the successful completion of cytokinesis, it is also crucial that dividing cells “counteract” the cellular tension exerted by their neighbors to stabilize the midbody (48). Thus, polarity and/or tension relative to neighboring cells can change cytokinesis outcomes, highlighting the unique adaptations and vulnerabilities of cell division in 3D tissue. Data being generated from a diverse set of model systems are demonstrating that disruption of this cell biology can alter lumen size and epithelial organization, both of which are phenotypes in *CIT*-affected organoids (49, 50). These findings provide important insight for explaining the 2D in vitro versus 3D in vivo phenotypic discrepancies described in both human and mouse models and how it is that cytokinesis defects can affect epithelial organization and integrity. Similar considerations of 3D cytoskeletal forces could be made in the study of CIT-K kinase molecular mechanisms in NPC genome integrity. Indeed, all models showed an increased amount of DNA damage, recapitulating the phenotype of affected individuals (30). In the future, they could provide new insight into the role of MCPH genes in mechanisms linking cytoskeletal organization to genome integrity (51, 52).

*CIT* was the first of a growing list of genes, including *KIF14*, *CEP55*, and *PPP1R12A*, that encode components of the cytokinesis machinery and are associated with severe brain-size abnormalities (7). *KIF14* (OMIM ID: 611279) and *CEP55* (OMIM ID: 610000), like *CIT*, are recessively inherited etiologies of microcephaly, whereas variants in *PPP1R12A* (OMIM ID: 602021) are responsible for a de novo dominant form of a holoprosencephaly spectrum that includes individuals with microcephaly (53–55). These proteins share similar subcellular localization at the midbody and functional overlap during abscission, yet a single pathogenic mechanism is not evident. CEP55 localizes to the bulge of the midbody and recruits endosomal sorting complexes required for transport (ESCRT) machinery, a conserved membrane remodeling system used in severing midbody flanks during abscission (56). During mitosis, KIF14, a microtubule motor, accumulates at the spindle midzone and localizes to the midbody in cells ready to undergo abscission (57). PPP1R12A accumulates at the central bulge of the midbody, regulates microtubule dynamics as a component of PP1β-MYPT1 phosphatase complex, and dephosphorylates the centralspindlin complex kinesin component MKLP1 (58). Pathogenic variants in each gene are associated with highly penetrant features that severely affect the brain, and some individuals also present with kidney abnormalities (53). Overall, with a minimal effect on other organ systems, these disorders punctuate the unique vulnerability of the developing brain to cytokinesis defects.

To better understand this unique vulnerability, several questions need to be answered. First, it is unknown which signaling pathways that coordinate cytokinesis are essential during brain development. Moreover, how the mammalian aNPC midbody forms at the apical membrane and interacts with cell junctions during cytokinesis and abscission has not, to our knowledge, been previously investigated in mouse or human models. These studies are further complicated by the limitations in evaluating the dynamics of cytokinesis cell biology in vivo. To tackle this problem, 3D live-cell imaging of sparsely labeled models can be used to track the long-term consequences of altering polarized cytokinesis. Finally, how the physical properties and composition of culture matrices, like Matrigel, constrain tissue and affect intracellular forces that affect 3D cytokinesis is yet to be determined. In conclusion, the identification and exploration of the aforementioned gaps in the field provide a compelling rationale for further research and investigation.

## Methods

### Sex as a biological variable.

The iPSC lines used in this work were derived from male and female individuals (Figure 3) as previously published (15). Moreover, our study examined male and female animals, and similar findings are reported for both sexes.

### Experimental animal work.

The mouse strain containing the FS *Cit* LOF variant (*Cit^FS/FS^*) on a C57BL/6 background was obtained from the UC Davis KOMP repository (*Cit^tm1a(KOMP)Wtsi^*). To generate CIT KI mice, we mutagenized *Cit* (c.376_377AA>GC; p.K126A) to generate the lysine 126 to alanine (K126A) substitution in the catalytic pocket in exon 3, using traditional ESC-based knockin technologies. This substitution totally inactivates the kinase domain and resembles pathogenic *CIT* variants found in affected individuals (14). We derived ESC clones harboring the correct homologous recombination event. Heterozygous (*Cit^+/KI^*) mice obtained from germline transmission of ESC clones were intercrossed to generate homozygous (*Cit^KI/KI^*) and control (*Cit^+/+^*) progeny. Age-matched WT littermates were used as controls.

### Behavioral characterization.

The behavioral characterization of P8–P9 mice, including evaluation of the maturity of ambulation, ataxic phenotype (i.e., spontaneous flipping on the back), vestibular reflexes, and coordination (i.e., surface righting reflex and negative geotaxis on a 45° inclined surface), has been described previously (59). For the balance beam test, we used a wood beam that was 1 cm wide × 100 cm long and suspended 12 cm above the bench. Two-month-old mice had to cross the beam to reach a cage enriched with toys. To familiarize the mice with the experimental apparatus, thus reducing the animal stress, the experiment was preceded by 3 days of acclimation. On the day of the test, the animals were allowed to acclimate to the behavioral room at least 15 minutes before the experiment. Both *Cit^KI/KI^* and *Cit^+/+^* mice were tested individually, and each animal was encouraged to traverse the beam at least 3 times. The test was repeated on 3 consecutive days. The test was recorded using a video camera and analyzed offline by an operator blinded to the genotype. The number of slips from the beam was measured to assess the mouse’s motor performance.

### In vitro kinase assay.

Recombinant glutathione S-transferase tagged myosin light chain (GST-MLC) was obtained from bacteria expressing the corresponding expression vector (gift of Kozo Kaibuchi, Nagoya University, Nagoya, Japan). Recombinant GST-CIT short kinase (GST-CIT-SK) was obtained using the BD Pharmingen baculovirus expression system by subcloning the mouse CIT-SK coding sequence (GenBank accession no. AF086823) in the pAcG2T Transfer Vector. In both cases, GST fusion proteins were isolated from total cell lysates of bacterial and insect cells through Glutathione Sepharose 4B (GE Healthcare Lifesciences) affinity chromatography according to the manufacturer’s specifications. For the in vitro kinase assay, recombinant proteins were incubated in 50 μL kinase buffer (50 mM HEPES, pH 7.4, 5 mM MgCl_2_, 3 mM MnCl_2_, plus 0.1 mM ATP and 10 mCi [γ-32P] ATP [6,000 Ci/mM, Amersham]) for 30 minutes at 30°C. The products were analyzed by 5% or 12.5% SDS-PAGE followed by autoradiography.

### Western blot analysis.

Tissues were lysed in RIPA buffer (1% NP40, 150 mmol/L NaCl, 50 mmol/L Tris-HCl pH 8, 5 mmol/L EDTA, 0.01% SDS, 0.005% sodium deoxycholate, Roche protease inhibitors, and PMSF) and homogenized in the same buffer with a pellet pestle (Z359971, MilliporeSigma). NPCs were washed twice with cold 1× PBS and then lysed with cold RIPA buffer. Cell lysates were collected and centrifuged at 14,000*g* at 4°C for 15 minutes. For immunoblots, equal amounts of protein from whole-cell lysates were resolved by SDS-PAGE and blotted to nitrocellulose or PVDF membranes.

### Mouse NPC isolation and culturing.

Embryonic brains were isolated from E12.5-timed pregnant mice. The lateral portion of the dorsal telencephalon was dissected, dissociated using a papain-based kit (Neural Tissue Dissociation Kit [P], Miltenyi Biotec), and plated at a concentration of 5 × 10^4^ cells/cm^2^ on polylysine/laminin-treated glass coverslips. Cells were analyzed 18 hours after plating. Culture medium comprised DMEM-F12, supplemented with 2% B27 without retinoic acid, 10 ng/mL EGF, and 40 ng/mL bFGF (all from Thermo Fisher Scientific). Cells were grown at 37°C in a humidified incubator with 5% CO_2_.

### Staining of mouse embryonic cortex and adult mouse brain.

Embryonic brains were dissected at E14.5 and fixed for 12–16 hours at 4°C in 4% paraformaldehyde (PFA). Postnatal mice were transcardially perfused with 4% PFA. After fixation, brains were equilibrated in 30% sucrose in PBS for 12–24 hours at 4°C, embedded with Tissue-TEK (OCT, Sakura Finetek), frozen in liquid nitrogen, and stored at –20°C. Sectioning was then performed with a cryostat to obtain 20 μm sections. Staining details can be found in the Supplemental Methods.

### Antibodies.

The following antibodies were used: mouse monoclonal anti-CIT (no. 611377), mouse monoclonal anti-Ki67 (no. 550609), and mouse monoclonal anti–N-cadherin (no. 610920) from Transduction Laboratories, BD Biosciences. Mouse monoclonal anti-vinculin (no. V9131), mouse monoclonal anti–α-tubulin (no. T5168), and rabbit polyclonal anti-Sox10 (HPA068898) were purchased from MilliporeSigma. Rabbit monoclonal anti-53BP1 (no. ab36823), rabbit polyclonal anti–Aurora B (no. ab2254), and mouse monoclonal anti–β-actin (no. ab6276) were obtained from Abcam. Rabbit polyclonal anti-cCASP3 (no. 9661S) and rabbit polyclonal anti-γH2AX (S139; 20E3; no. 2577) were from Cell Signaling Technology. Rabbit polyclonal anti-BLBP (ABN14) was purchased from MilliporeSigma. Rat anti-BCL11B (A48270) was from Invitrogen, Thermo Fisher Scientific. Mouse monoclonal anti-MKLP1 (sc-390113) and goat polyclonal anti-DCX (sc-8066) were purchased from Santa Cruz Biotechnology. Mouse monoclonal anti-SOX2 (no. MAB2018) was from R&D Systems. Neuronal cells were detected by rabbit anti-calbindin (1:1,000, SWANT, CB-38a). Click-it TUNEL (no. C10619) from Thermo Fisher Scientific was used for cell death detection in mice and organoids.

### hPSC culturing.

The iPSC lines used in this work were previously described (15). Briefly, mycoplasma-free fibroblast lines from affected individuals, carrier individuals, and healthy unrelated controls were episomally reprogrammed. On average, 15 iPSC lines were established from the reprogramming of 1 × 10^6^ fibroblasts. Six lines were expanded from each genotype and characterized. The pluripotent and genomic integrity of these lines were characterized for morphology and growth rate, karyotype to rule out numerical and structural chromosomal abnormalities, and the expression of pluripotency markers (NANOG, TRA-1-81, LIN28, and TRA-1-60). Low-passage (p24) H9 hESCs (lot no. WB0090) were obtained from WiCell. Short tandem repeat analysis and sterility and quality tests were performed by WiCell. Chromosomal microarray confirmed that no hPSC line used had copy number variants (CNVs) associated with CNV syndromes defined according to the Decipher database. hPSCs were maintained in mTeSR1 medium (no. 85850, STEMCELL Technologies) on Matrigel-coated dishes (no. 354234, Corning) in a cell incubator at 37°C with 5% CO_2_. Stem cells were chemically dissociated at 37°C using Versene (no. 15040066, Invitrogen, Thermo Fisher Scientific) or were mechanically dissociated and then resuspended in mTeSR1 with the addition of Y27632 ROCK inhibitor (Tocris) at a final concentration of 10 μM for 24 hours to promote survival when cells were passaged. The mTeSR1 medium was changed daily. Cells were not used past passage 50.

### hESC CIT^FS/FS^ generation by CRISPR/Cas9 genome editing.

*CIT^FS/FS^* cell lines were generated by CRISPR/Cas9 genome editing to obtain a homozygous *CIT* FS genotype (*CIT* NM_007174.3: c.312_318del, p.C105Tfs*8) from newly thawed low-passage H9 cells. A guide RNA expression vector was generated using annealed oligonucleotides (forward, 5′-CACCGTCAGAAGtCTTGTAGGTTG-3′; reverse, 5′-AAACCAACCTACAAGACTTCTGAC-3′) designed to target exon 4 of *CIT*. The annealed oligonucleotides were ligated into the Bbs1 restriction–digested pX330. Subsequent transformation was accomplished using chemically competent Stbl3 *E. coli* (Invitrogen, Thermo Fisher Scientific), and the cell suspension was plated on Lennox broth agar plates supplemented with ampicillin. Plates were incubated overnight for 16 hours at 37°C. Colonies were selected and inoculated into Lennox broth with ampicillin to be later extracted by Mini-prep (QIAGEN). The extracted plasmids were screened with restriction digest and Sanger sequencing using the U6 forward primer 5′-GAGGGCCTATTTCCCATGATTCC-3′. Plasmids containing the insertions were further amplified and extracted using the Nucleobond Xtra Midi Plus EF kit (Macherey-Nagel) for experiments. Electroporation was performed using the Lonza Human Stem Cell Nucleofector Kit 2 (Lonza) according to the manufacturer’s instructions. Electroporated cells were transferred to a Matrigel-coated dish containing mTeSR1 supplemented with Y27632 ROCK inhibitor (Tocris) and Normocin (InvivoGen). A few days later, the cells were then diluted into Matrigel-coated, 96-well plates for screening. Wells with individual colonies were screened by Sanger sequencing using primers to amplify exon 4 of *CIT* (forward, 5′-TGGGGCTAACTCTTGCATCT-3′; reverse, 5′-CACTCCGGTACAGAAAGTGGA-3′). Edited cell lines were analyzed by chromosomal microarray to confirm genomic integrity and Western blotting to confirm the absence of CIT-K protein expression.

### Generation of hPSCs with stable expression of Lifeact-GFP and H2B-mCherry fluorescence reporters.

hPSCs were stably labeled with H2B-mCherry and Lifeact-GFP using the PiggyBac transposase method, which allows for random genomic integration of DNA sequences containing inverted terminal repeat sequences (34). hPSCs were transfected at a low-passage number using Lipofectamine Stem reagent (Invitrogen, Thermo Fisher Scientific) and Opti-MEM I Reduced Serum medium (Invitrogen, Thermo Fisher Scientific). Three plasmids were transfected at equal parts: pCAG:H2B-Cherry, pCAG:Lifeact-GFP, and pCAG:PBase expressing the PiggyBac transposase, provided by Orly Reiner at the Weizmann Institute of Science (Rehovot, Israel). Mechanical passaging was used to select for cells expressing both H2B-mCherry and Lifeact-GFP.

### Neural differentiation.

hPSCs were differentiated into NPCs and dorsal forebrain organoids using dual inhibition of SMAD signaling as previously described (31). The following media were used during the differentiation protocol: (a) N2 plus dual SMAD inhibition medium containing 1:100 N2 supplement (no. 17502048, Thermo Fisher Scientific), 1 μM dorsomorphin (Tocris), 2 μM A83-01 (Tocris) in DMEM/F12 (no. 11320033, Gibco, Thermo Fisher Scientific); (b) N2 plus B27 plus dorsomorphin medium containing 1:200 N2 supplement, 1:100 B27 supplement (no. 17504044, Thermo Fisher Scientific), 1 μM dorsomorphin, 20 ng/mL basic FGF (bFGF) in DMEM/F12; and (c) N2 plus B27 medium containing 1:200 N2 supplement, 1:100 B27 supplement, 20 ng/mL bFGF in DMEM/F12.

### Generation and maintenance of hPSC aggregates (0–7DD).

hPSCs were chemically dissociated at 37°C using Versene or StemPro Accutase Cell Dissociation Reagent (no. A1110501, Gibco, Thermo Fisher Scientific) and resuspended in mTeSR1 with added Y27632 ROCK inhibitor at a final concentration of 10 μM. Cells were counted and diluted to a concentration of 2 × 10^4^ cells/mL. The cell suspension (30 μL), containing approximately 600 cells, was dispensed in each well of an ultra-low cell attachment 96-well plate with a V-shaped bottom (no. MS-9096VZ S-Bio) to encourage cell aggregation. This step is considered day 0 of differentiation (0DD). Neural induction was initiated 36 hours later (1.5DD) by adding 150 μL N2 plus dual SMAD inhibition medium to each well, and on 3DD, aggregates were transferred to ultra-low cell attachment 6-well plates to continue growth in suspension. Media were changed every 48 hours. Aggregates were inserted into preconstructed compartments at 7DD.

### Construction of microfabricated devices, assembly, and confocal analysis.

Device fabrication and assembly were adapted from established protocols (35, 60). Details can be found in the Supplemental Methods. Confocal imaging was conducted on 21DD, 28DD, and 35DD using a Nikon X1 Yokogawa spinning-disk confocal and a Tokai Hit incubated stage to maintain device incubation. Details can be found in the Supplemental Methods.

### NPC and organoid differentiation.

hPSC aggregates were generated and maintained (0–7DD) as described above. On 7DD, the medium was changed to N2 plus B27 plus dorsomorphin, and aggregates were transferred to a 6-well plate (Corning) coated with Matrigel using a 200 μL pipette with a razor-cut tip to minimize shearing forces on the aggregates (~12–15 aggregates/well). Extra medium was then added to reach 2 mL N2 plusB27 plus dorsomorphin per well. Media were changed every 48 hours. On 14DD, NPCs were generated by gently detaching neural rosettes from Matrigel using a p200 tip, followed by chemical dissociation using Accutase (Invitrogen, Thermo Fisher Scientific). Dissociated cells were plated on 15 μg/mL poly-l-ornithine (MilliporeSigma) and 10 μg/mL laminin-coated (MilliporeSigma) dishes. For dorsal forebrain organoid generation, neural rosettes were gently detached from Matrigel using a p200 tip, and the neural tissue was grown in suspension under constant rotation at 95 rpm. The N2 plus B27 medium was used on 14DD, with a medium change every other day until 35DD.

### Organoid cryopreservation, embedment, and sectioning.

Organoids were fixed in 4% PFA overnight before beginning cryopreservation. Organoids were saturated in a 15% sucrose solution overnight or until tissue sank to the bottom of a conical tube before applying the final 30% sucrose solution. Once tissue was sufficiently cryopreserved, the organoids were placed in a plastic cassette containing OCT medium (Thermo Fisher Scientific) and frozen using –60°C to –70°C 2-methyl butane. OCT-containing organoid blocks were sectioned at 13 μm using a cryostat (Leica Biosystems).

### Immunofluorescence on organoids.

Cryopreserved sections (13 μm thick) of cerebral organoid tissue adhered on slides (Thermo Fisher Scientific) were used for immunofluorescence. A subset of slides to be stained with CIT, MKLP1, BCL11B, cleaved caspase 3, or γH2AX were treated with sodium citrate (10 mM) antigen retrieval prior to staining. Slides were washed 3 times with 1× PBS for 5 minutes. Tissue was then permeabilized with 0.1% Triton X-100 for 5 minutes and subsequently washed once with 1× PBS for 5 minutes before blocking buffer was implemented. Organoid sections were incubated with blocking buffer (0.1% Triton X-100, 1% BSA, and 5% NDS) for 1 hour at room temperature. Primary antibodies were diluted in blocking buffer and incubated at 4°C overnight. Sections were then exposed for 1–2 hours at room temperature to secondary antibodies conjugated with Alexa Fluor (Invitrogen, Thermo Fisher Scientific), and finally incubated with Hoechst (Invitrogen, Thermo Fisher Scientific) diluted at 1:10,000 in PBS for 5 minutes. Coverslips were mounted on the glass slides using Prolong Gold (Invitrogen, Thermo Fisher Scientific) overnight before imaging.

### Image processing and data analysis.

Details can be found in the Supplemental Methods.

### Statistics.

Statistical analyses were performed using Excel, version 16 (Microsoft) and GraphPad Prism, version 10 (GraphPad Software). An unpaired, 2-tailed Student’s *t* test was used to compare 2 groups, and a 1-way or 2-way ANOVA was used for multiple-group comparisons followed by Bonferroni or Holm-Šidák post hoc analysis. A Mann-Whitney *U* test was used to analyze γH2AX and 53BP1 foci and time spent from anaphase to G_1_ ascension during cell division. A Fisher’s exact probability test was used for percentage distribution. Data are shown as the mean values of at least 3 independent experiments unless otherwise noted and the mean ± SEM. A P value of less than 0.05 was considered statistically significant.

### Study approval.

For iPSC lines, individuals were enrolled according to protocols approved by the IRBs at the affiliated institutions (University of Michigan, Ann Arbor, Michigan, USA and UCSD, San Diego, California, USA), as previously described (15). In all cases, the procedures followed were in accordance with the ethics standards of the respective institution’s committee on human research and were in keeping with international standards. Written informed consent was obtained prior to participation. The animal studies were designed according to the guidelines of the NIH, the European Communities Council (2010/63/EU), and the Italian Law for Care and Use of Experimental Animals (DL26/2014) under permission number 1128/2020-PR, released on November 16, 2020, from the Italian Ministry of Health, Department of Public Veterinary Health. The animal studies were also approved by the Italian Ministry of Health and the Bioethical Committee of the University of Turin.

### Data availability.

The original contributions presented in the study are included in the article and supplemental material. Supporting data values associated with the main article and supplemental material are included in the Supporting Data Values file, with separate tabs for each applicable figure panel. Further inquiries can be directed to the corresponding authors.

## Author contributions

GP, AM, FDC, SLB conceived of and designed the study. AM, GP, FB, ET, OR, and RYT developed the methodology. GP, AM, GI, RP, ERP, AF, GEB, EB, ML, SS, HFM, SW, NG, MC, and JE acquired data. GI, AM, GP, ERP, and EB performed data analysis. GP, AM, GI, ERP, EB, OR, AB, FDC, and SLB interpreted the data. GP, AM, GI, ERP, FDC, and SLB wrote the manuscript. All authors contributed to the article and approved the submitted version. GP is the first author in the list of shared first authors, as he initiated the project and performed animal experiments;AM is the second author in the list of shared first authors, as she initiated the project and performed the majority of the organoid experiments; and GI is the third author in the list of shared first authors, as she completed the analysis to bring this manuscript to completion.

## Supplementary Material

Supplemental data

Unedited blot and gel images

Supplemental video 1

Supplemental video 2

Supplemental video 3

Supplemental video 4

Supplemental video 5

Supplemental video 6

Supporting data values

## Version 1 09/24/2024

In-Press Preview

## Version 2 11/01/2024

Electronic publication
