# Supplementary material for: Modeling primary microcephaly with human brain organoids reveals fundamental roles of CIT kinase activity
Source: J Clin Invest. 2024 Nov 1;134(21):e175435. doi: 10.1172/JCI175435 (PMC11527453; doi:10.1172/JCI175435)
Supplement: Supplemental data [file jci-134-175435-s119.pdf]

Supplemental material

Modeling primary microcephaly with human brain organoids reveals fundamental roles of CIT kinase activity

Gianmarco Pallavicini* [1,2], Amanda Moccia* [3], Giorgia Iegiani* [1,2], Roberta Parolisi[1,2], Emily R. Peirent [4], Gaia Elena Berto[1,2], Martina Lorenzati[1,2], Rami Y. Tshuva[5], Alessia Ferraro [1,2], Fiorella Balzac[6], Emilia Turco[6], Shachi Salvi[3], Hedvig F. Myklebust[3], Sophia Wang[3], Julia Eisenberg[3,7], Maushmi Chitale[3], Navjit Girgla[3], Enrica Boda[1,2], Orly Reiner [5], Annalisa Buffo[1,2], Ferdinando Di Cunto[$1,2] and Stephanie L. Bielas[$3,4,7]

1. Neuroscience Institute Cavalieri Ottolenghi, Turin, Italy.

2. Department of Neuroscience 'Rita Levi Montalcini', University of Turin, Italy

3. Department of Human Genetics, University of Michigan Medical School, Ann Arbor, MI, USA

4. Neuroscience Graduate Program, University of Michigan Medical School, Ann Arbor, MI, USA

5. Departments of Molecular Genetics and Molecular Neuroscience, Weizmann Institute of Science, Rehovot, Israel

6. Department of Molecular Biotechnology and Health Sciences, University of Turin, Italy

7.Department of Pediatrics, University of Michigan Medical School, Ann Arbor, MI, USA

* These authors contributed equally to this work

$ Corresponding author:

Ferdinando Di Cunto (Ferdinando Di Cunto, Neuroscience Institute Cavalieri Ottolenghi Regione Gonzole, 10, 10043 Orbassano (TO), Italy,+39-011-6706616, ferdinando.dicunto@unito.it)

Stephanie L. Bielas (Stephanie L. Bielas, Department of Human Genetics, University of Michigan Medical School, 3703 Med Sci II, 1241 E. Catherine St., Ann Arbor, Michigan, 48109-5618, USA, 1-734-647-8890, sbielas@med.umich.edu)

The authors have declared that no conflict of interest exists.

Supplemental figures

Figure S1 Generation of $Cit^{KI/KI}$ mice

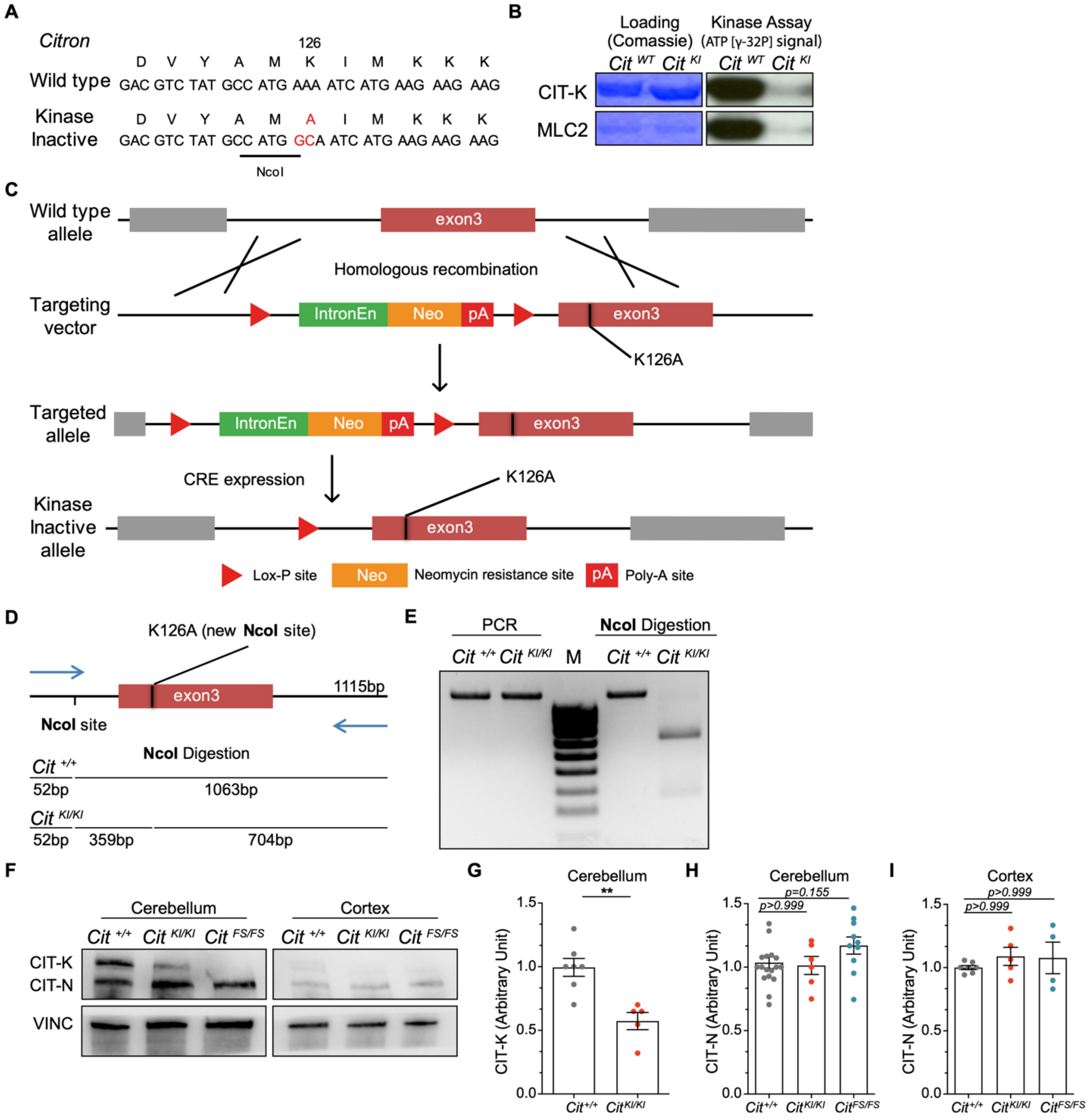

(**A**) Design of the *Cit* kinase inactive allele. Using traditional ES cell-based knock-in technologies, the c.376_377AA>GC; p.K126A substitution was planned. The resulting NcoI restriction site in mutant allele is shown.

(**B**) Cit^{WT} and Cit^{KI} purified mouse proteins were incubated with MLC2 in the presence of [γ-32P] ATP for a kinase assay. The reactions were then analyzed by SDS-PAGE and autoradiography (right panel). Coomassie-stained gel is shown for loading control (left panel).

(**C**) Schematics of the recombination strategy adopted to generate Cit^{KI} allele.

(**D**) Schematics of the PCR and restriction digestion strategy used to discriminate $\mathit{Cit}^{\mathrm{WT}}$ and Cit^{KI} alleles in mouse genotyping.

(**E**) Example of mouse genotyping performed with the strategy summarized in (D), showing the results of two littermate mice obtained from $\mathit{Cit}^{+/KI}$ intercrossing (M= marker).

(**F**) Western blot analysis of total lysate from P4 cerebellum and cortex obtained from mice of the indicated genotypes. CIT expression was analyzed, and the internal loading control was vinculin (VINC).

(**G-I**) Quantification of CIT-K and CIT-N relative abundance in P4 cerebella and cortex of the indicated genotypes. The absolute intensity of CIT-K and CIT-N bands in $\mathit{Cit}^{+/+}$, $\mathit{Cit}^{KI/KI}$ and $\mathit{Cit}^{FS/FS}$ samples was normalized on the corresponding vinculin intensity and the resulting averages were divided by the average of $\mathit{Cit}^{+/+}$ values. Each dot represents an independent replicate.

Error bars, SEM. **P<0.01; unpaired Student's t-test (G) and one-way ANOVA followed by Bonferroni's post-hoc analysis (H-I).

<u>Figure S2</u> Morphological and behavioral characterization of *Cit*$^{KI/KI}$ mice

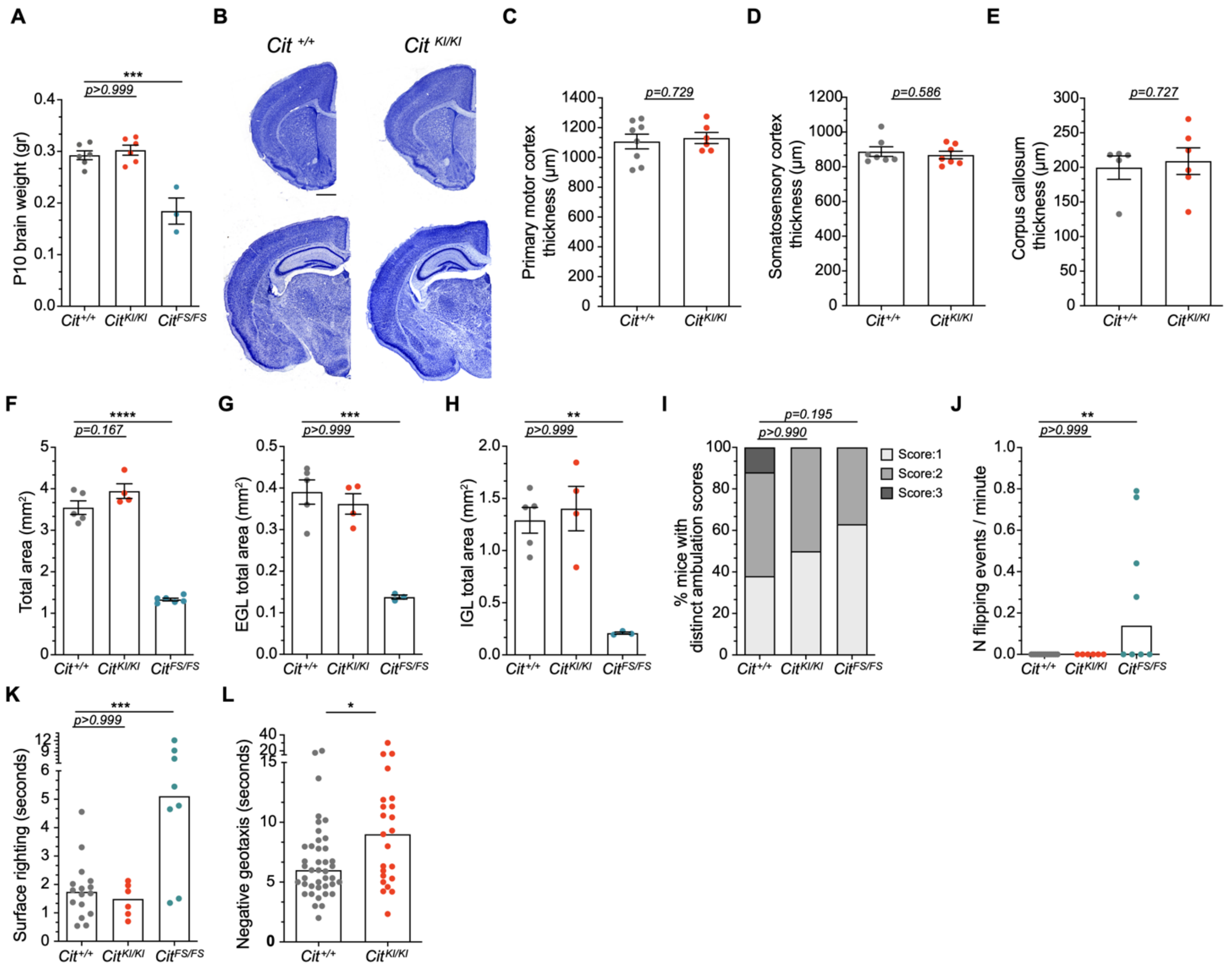

(**A**) Measurement of *Cit* $^{+/+}$, *Cit*$^{KI/KI}$ and *Cit*$^{FS/FS}$ P10 brain weight.

(**B**) Cresyl Violet staining of rostral (upper) and caudal (lower) coronal sections of *Cit* $^{+/+}$ and *Cit*$^{KI/KI}$ P10 brains. Scale bar = 2mm.

(**C**) Quantification of primary motor cortex thickness of P10 *Cit* $^{+/+}$ and *Cit*$^{KI/KI}$ mice.

(**D**) Quantification of somatosensory cortex thickness of P10 *Cit* $^{+/+}$ and *Cit*$^{KI/KI}$ mice.

(**E**) Quantification of corpus callosum thickness of P10 *Cit* $^{+/+}$ and *Cit*$^{KI/KI}$ mice.

(**F**) Quantification of cerebellum total area of P10 *Cit* $^{+/+}$, *Cit*$^{KI/KI}$ and *Cit*$^{FS/FS}$ mice.

(**G**) Quantification of cerebellum external granule layer (EGL) total area of P10 *Cit* $^{+/+}$, *Cit*$^{KI/KI}$ and *Cit*$^{FS/FS}$ mice.

(**H**) Quantification of cerebellum internal granule layer (IGL) total area of P10 *Cit* $^{+/+}$, *Cit*$^{KI/KI}$ and *Cit*$^{FS/FS}$ mice.

(**I**) Quantification of *Cit* $^{+/+}$, *Cit*$^{KI/KI}$ and *Cit*$^{FS/FS}$ mice ambulation score using the following scale: 0 = no movement, 1 = crawling with asymmetric limb movement, 2 = slow crawling but symmetric limb movement, and 3 = fast crawling/walking.

(**J**) Quantification of *Cit* $^{+/+}$, *Cit*$^{KI/KI}$ and *Cit*$^{FS/FS}$ mice spontaneous flipping events per minute.

(**K**) Quantification of *Cit* $^{+/+}$, *Cit*$^{KI/KI}$ and *Cit*$^{FS/FS}$ mice surface righting reflex.

(**L**) Quantification of *Cit* $^{+/+}$ and *Cit*$^{KI/KI}$ mice time needed to perform negative geotaxis on a 45° inclined surface.

Error bars, SEM. *P<0.05,**P<0.01, ***P<0.001; ****P<0.0001; unpaired Student's t-test (C,D,E and L), one-way ANOVA test followed by Bonferroni's post-hoc analysis (A,F,G,H,J and K) and Fisher exact probability test for percentage distribution (I). Each dot represents an independent animal.

Figure S3 ***Cit*$^{KI/KI}$ P10 cerebella show increased DNA damage but not apoptosis**

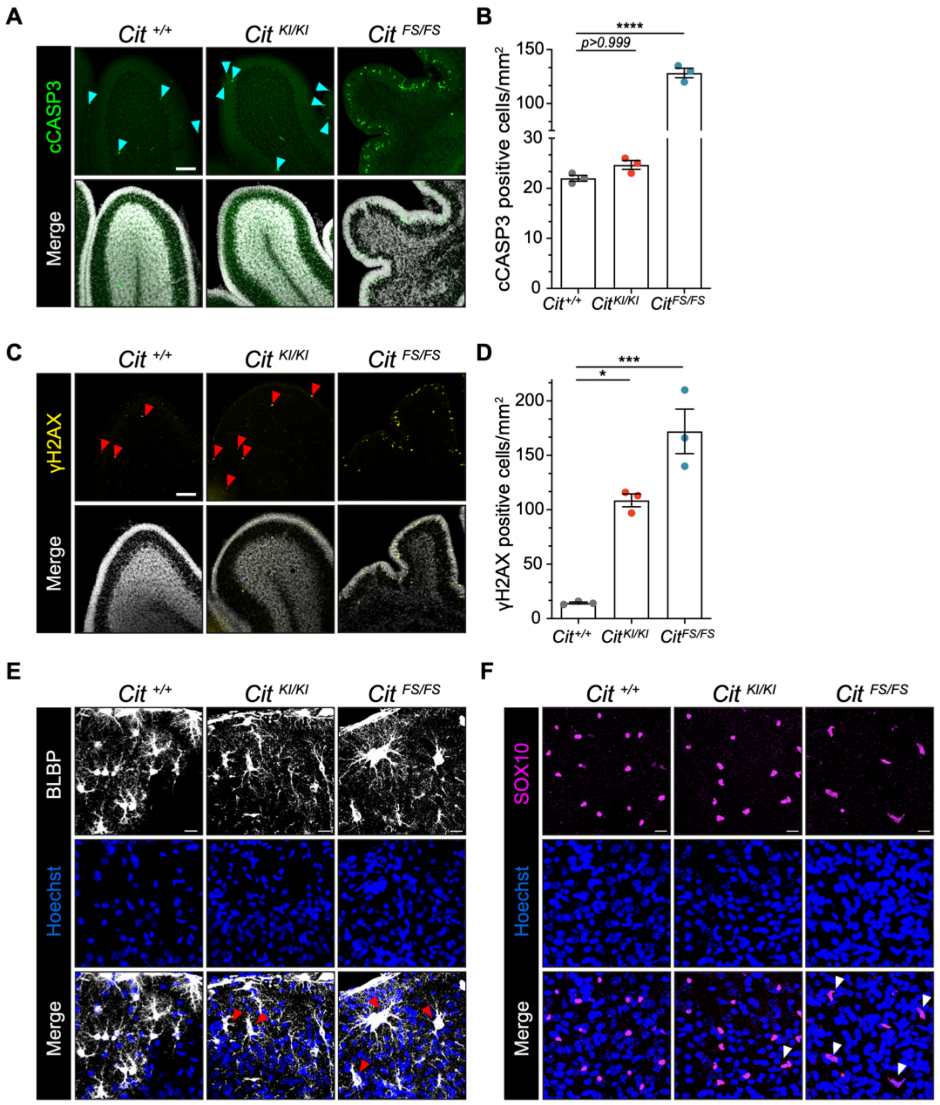

(**A**) Immunofluorescence analysis of P10 cerebella (vermis) for apoptotic marker cCASP3 (green) and Hoechst (gray). Blue arrowheads indicate positive cells. Scale bar = 25μm.

(**B**) Quantification of cCASP3 positive cells in (A). Every dot represents an independent animal, and 3 fields per animal were analyzed.

(**C**) Immunofluorescence analysis of P10 cerebella (vermis) for DNA damage marker γH2AX (yellow) and Hoechst (gray). Red arrowheads indicate positive cells. Scale bar = 25μm.

(**D**) Quantification of γH2AX positive cells in (C). Every dot represents an independent animal, and 3 fields per animal were analyzed.

(**E**) Representative images of BLBP-positive astrocytes (white) and Hoechst (blue) in P10 cortex of *Cit*$^{+/+}$, *Cit*$^{KI/KI}$ and *Cit*$^{FS/FS}$ mice. Red arrowheads indicate binucleated cells. Scale bars = 25μm.

(**F**) Representative images of SOX10-positive oligodendroglial lineage (magenta) and Hoechst (blue) in P10 cortex of *Cit*$^{+/+}$, *Cit*$^{KI/KI}$ and *Cit*$^{FS/FS}$ mice. Red arrowheads indicate binucleated cells. Scale bars = 25μm.

Error bars, SEM. *P<0.05, ***P<0.001; ****P<0.0001; one-way ANOVA test followed by Bonferroni's post-hoc analysis.

Figure S4 Characterization of organoid cell type composition

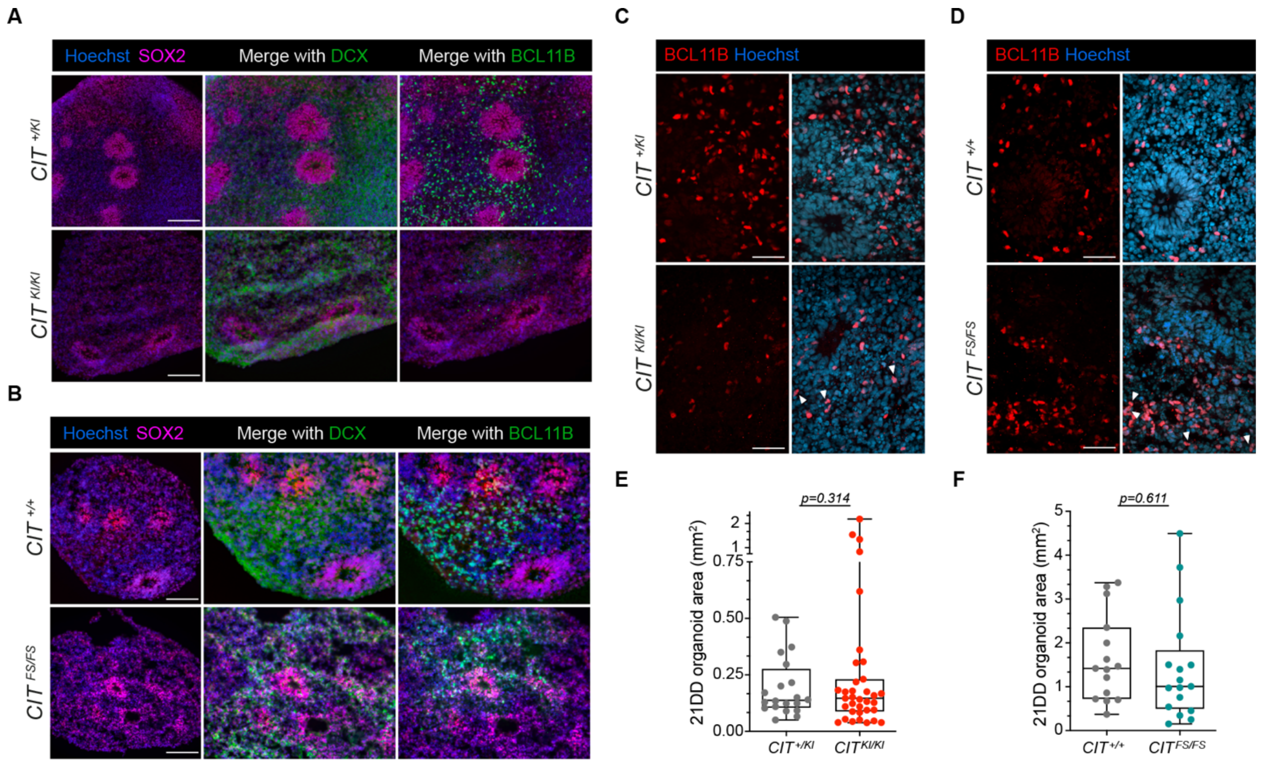

Images S4 A-F are generated from organoids differentiated from fluorescent reporter-free hPSC lines. (**A**) Representative images of $CIT^{+/KI}$ and $CIT^{KI/KI}$ 35DD organoids immunostained with SOX2 (magenta) to label progenitors, DCX (green) to label immature postmitotic progeny, BCL11B (green) to label deep-layer cortical neurons and Hoechst (blue). Scale bars = 50µm

(**B**) Representative images of $CIT^{+/+}$ and $CIT^{FS/FS}$ 35DD organoids immunostained with SOX2 (magenta) to label progenitors, DCX (green) to label immature postmitotic progeny, BCL11B (green) to label deep-layer cortical neurons and Hoechst (blue). Scale bars = 50µm

(**C**) Representative images of $CIT^{+/KI}$ and $CIT^{KI/KI}$ 35DD organoids immunostained with BCL11B (red) and Hoechst (blue). Arrowheads indicate binucleated cells. Scale bars = 50µm

(**D**) Representative images of $CIT^{+/+}$ and $CIT^{FS/FS}$ 35DD organoids immunostained with BCL11B (red) and Hoechst (blue). Arrowheads indicate binucleated cells. Scale bars = 50µm

(**E-F**) Quantification of 21DD organoid area of $CIT^{KI/KI}$ (E) and $CIT^{FS/FS}$ (F) relative to their controls. Error bars, SEM. Unpaired two-tailed Student's t-test. Each dot represents an organoid at 21DD.

<u>Figure S5</u> *CIT*$^{KI/KI}$ and *CIT*$^{FS/FS}$ organoids show enlarged apical endfeet surface area

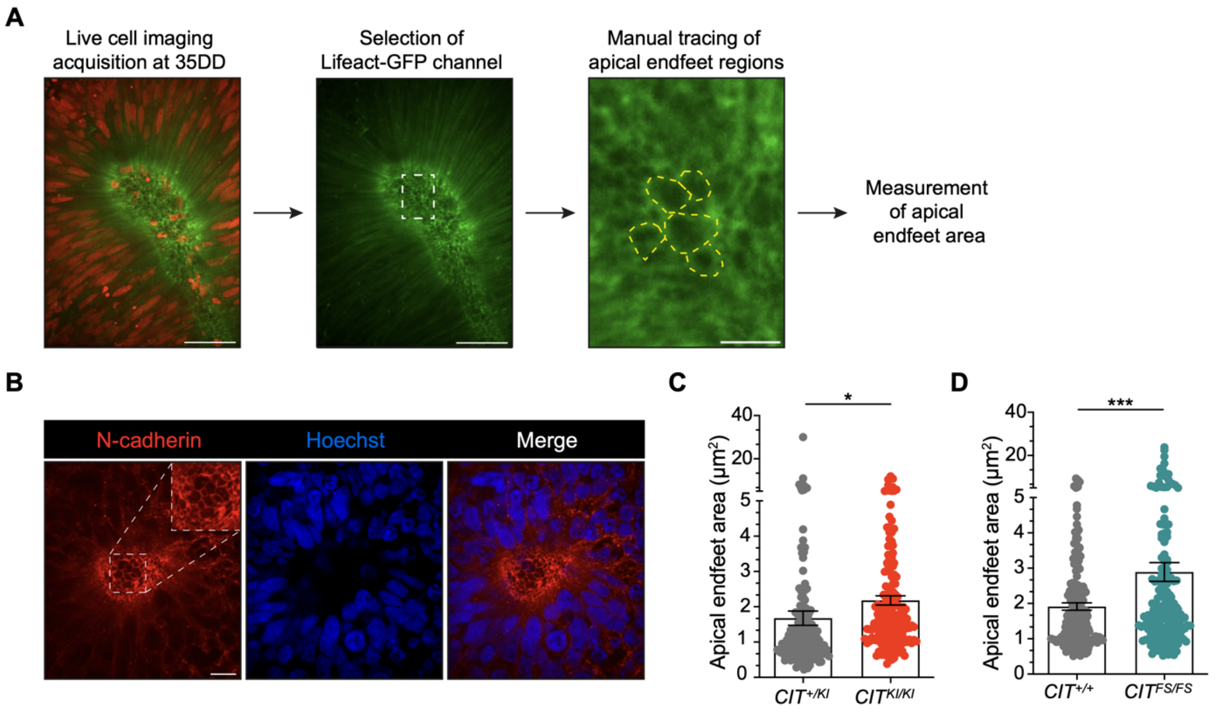

(**A**) Scheme for measuring of apical endfeet surface area in the central lumen of organoids differentiated from fluorescent reporter labeled hPSCs. H2B-mCherry (red) and Lifeact-GFP (green).

(**B**) Representative images of a 35DD organoid, differentiated from fluorescent reporter-free hPSC lines, immunostained with N-cadherin (red) to mark the apical surface and Hoechst (blue). Scale bars = 10μm

(**C**) Quantification of apical endfeet surface area of *CIT*$^{+/KI}$ and *CIT*$^{KI/KI}$ in 35DD organoids.

(**D**) Quantification of apical endfeet surface area of *CIT*$^{+/+}$ and *CIT*$^{FS/FS}$ in 35DD organoids.

Error bars, SEM. *P<0.05, ***P<0.001; Unpaired two-tailed Student's t-test.

<u>Figure S6</u> Apical surface of *CIT*- affected rosettes show an increase in midbody mislocalization and altered cell division plane

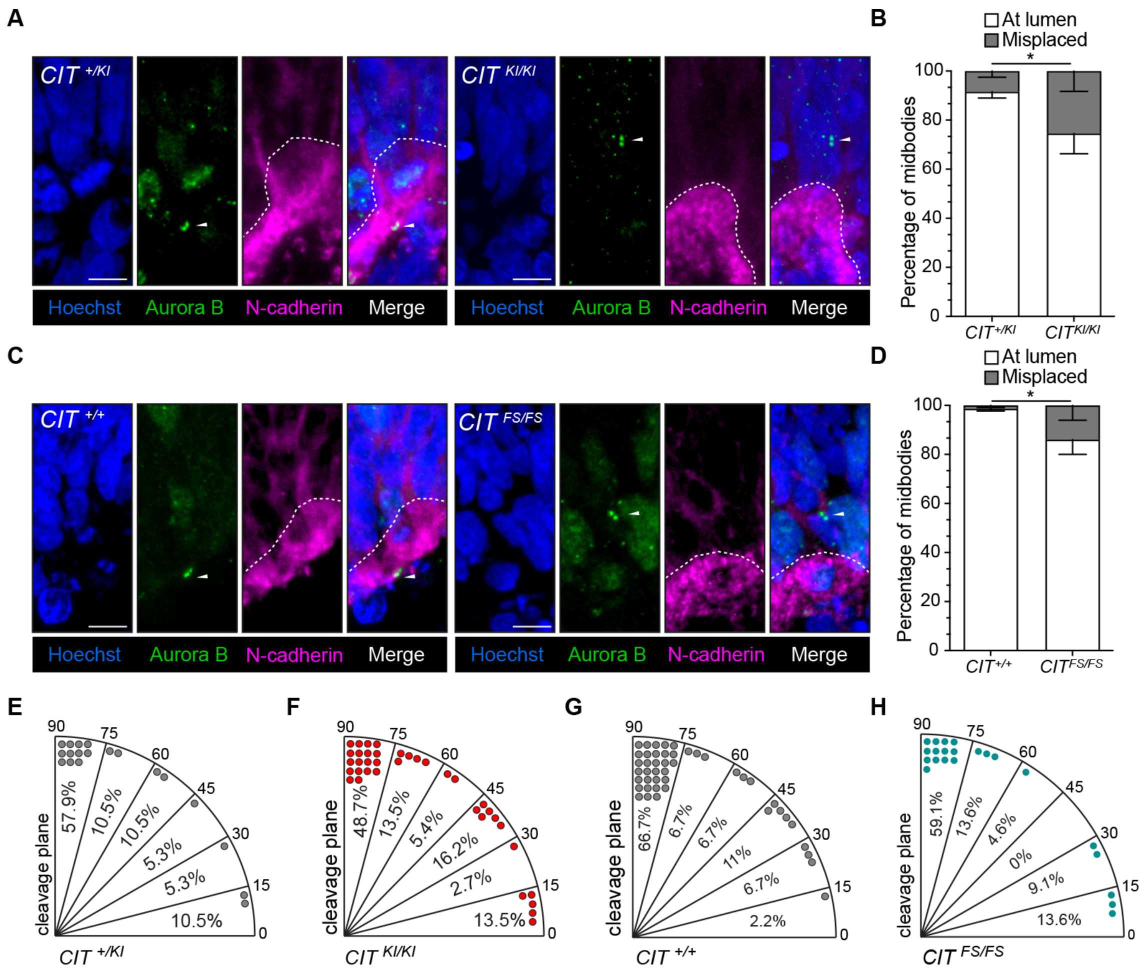

(**A**) Representative images and (**B**) quantification of midbodies per central lumen in *CIT*$^{+/KI}$ and *CIT*$^{KI/KI}$ 35DD organoids differentiated from fluorescent reporter free hPSC lines. Immunostaining with N-cadherin (magenta) marked the apical surface and Aurora B (green) marked the midbody arms (white arrowheads) apically aligned and misaligned. DNA stained with Hoechst (blue). Quantification from 3 independent organoid preparations. Scale bars = 10µm.

(**C**) Representative images and (**D**) quantification of midbodies as outlined above for *CIT*$^{+/+}$ and *CIT*$^{FS/FS}$. Quantification from 3 independent organoid preparations. Scale bars = 10µm.

(**E-H**) Measurement of cleavage plane angle of mitotic aNPCs from live imaging in *CIT*$^{+/KI}$ (E), *CIT*$^{KI/KI}$ (F), *CIT*$^{+/+}$(G) and *CIT*$^{FS/FS}$ (H) 35DD organoid neural rosettes. Each dot indicates a single dividing cell. Quantification for a minimum of 2 independent compartment preparation per

genotype. Compartments each contain 9 or fewer organoids per preparation. Error bars, SEM.

*$P<0.05$, Fisher Exact Probability Test for percentage distribution.

Supplemental methods

Staining of mouse embryonic cortex and adult mouse brain

For staining, cryo-sections were subjected to antigen retrieval using 10mM Na Citrate (pH 6.5) and 1% Glycerol in PBS. Sections were then permeabilized with 0.3% Triton X-100 in PBS for 30 minutes, quenched with 0.1M glycine for 30 minutes and incubated overnight at 4°C with primary antibody.

For P4 and P10 mice, 40µm-thick cryostat sections were collected in PBS. For histological analysis, the sections were stained with 0.5% cresyl violet, treated with alcohol for rapid color separation, permeabilized with xylene, and mounted on microscope slides with Neo-Mount (Sigma-Aldrich, Saint Louis, MS, USA). For immunohistochemical analysis, after 3 washes in PBS, sections were incubated in blocking buffer (2% Normal Donkey Serum (NDS), 1% Bovine Serum Albumin (BSA), 1% Triton X-100 in PBS) for at least 1 hour, then incubated overnight at 4°C with primary antibody. For Cleaved Caspase3 and γH2AX- staining, the sections were rinsed in PBS and antigen retrieval was performed using Citric Acid at 90°C before the blocking step. Sections were then exposed for 2 h at room temperature to secondary anti-rabbit antibodies conjugated with Alexa Fluor 546 or Alexa Fluor 488 (Invitrogen, Waltham, MA, USA), anti-mouse conjugated with Cy3 (1:500; Jackson ImmunoResearch, West Grove, PA, USA). Hoechst (Invitrogen) was used to label nuclei. After processing, sections were mounted on microscope slides with Tris-glycerol supplemented with 10% Mowiol (Calbiochem, LaJolla, CA).

Construction of microfabricated devices

Device fabrication and assembly were adapted from established protocols (35, 58). The bottom of a 6cm tissue culture dish (USA Scientific) was patterned with eleven 1.5mm holes. Holes were patterned using a custom 3D printed cassette (kind gift of Dr. David Burke, Ann Arbor, MI) locking the dish bottom in place while a manual drill with a 1.5mm drill bit slowly drilled through the polystyrene material. Dishes were cleaned with 70% ethanol inside a tissue culture hood and placed under UV for ten minutes to promote sterility. Then, a 5µL stripe of UV-curable adhesive NOA81 (Norland Products) was placed above the prepatterned holes on the bottom surface of the pre-patterned tissue culture dish. Using a double-sided razor, the UV-curable adhesive was spread

uniformly across the holes. The adhesive was partially cured using a 20W UV flashlight (Shenzhen Lightfe Lighting Co., Ltd) on a setting of low brightness (3W, 130mA) for eight seconds. A microporous circular polycarbonate membrane 13μm in diameter with a 0.4μm pore size (Cytiva Whatman Cyclopore PC Polycarbonate Membrane Filters) was applied to this same bottom surface over the nine medial holes. Gentle pressure on the membrane onto the polystyrene ensured good contact of the two materials. Full curation of the adhesive was then conducted with the UV flashlight on a setting of moderate brightness (10W, 400mA) for two minutes to the fabricated dish bottom. The dish bottom was then reunited with a dish top and placed in a 10cm petri dish with the membrane facing upwards. The petri dish served as a secondary container for the next step to evaporate the remaining adhesive in an oven set at 80°C for two hours. Coverslips were prepared in advance using a circular polydimethylsiloxane (PDMS) stamp 20mm in diameter with a thickness of 150um. The PDMS stamp was kindly provided by Dr. Orly Reiner at the Weizmann Institute of Science. A 24 x 24 mm No.1 square glass coverslip (Matsunami) was placed on the center of the PDMS stamp. Gentle downward pressure on the coverslip ensured good contact of the two materials. The UV-curable adhesive was applied to an edge of the glass coverslip and allowed to enter the 150μm gap by capillary flow. This process was repeated until all four coverslip sides were filled with adhesive while the circular center remained intact. Partial curation of the adhesive was then conducted using the UV flashlight on a setting of low brightness (3W, 130mA) for three seconds. The PDMS stamp was then gently peeled from the adhesive coated coverslip.

Microfabricated device assembly (7DD)

On 7DD, aggregates were transferred into a new dish containing N2+B27+Dorsomorphin medium. Nine aggregates were placed on an adhesive coated coverslip (adhesive facing up). Each aggregate was collected in 1μL of medium and placed directly on the glass in a pattern that mirrored the nine medial patterned holes on the device. Once completed, the coverslip was inverted using tweezers and placed on top of the device with the circular center covering all eleven patterned holes. Gentle force on top of the coverslip edges using the back of the tweezers ensured good contact of the glass and tissue culture dish. The edges of the coverslip were sealed with a

10μL stripe of UV-curable adhesive. The inverted dish containing the aggregates were placed over an opaque mask to block the tissue from final curation with UV. The device was cured using the UV flashlight on a setting of moderate brightness (10W, 400mA) for two minutes. The device was turned upright and reunited with its dish top. 200μL of N2+B27+Dorsomorphin medium was added on top of the nine holes to allow diffusion into the chamber containing the aggregates. The device was placed in a petri dish on top of added PDMS squares to prevent scratches to the coverslip. The secondary container with the device was then placed in the cell incubator for 10-20 minutes to allow the chamber to fill with medium. Once complete, the device was then filled with 3mL of N2+B27+Dorsomorphin medium and placed back into cell incubator.

Matrigel embedment of the device (9DD) and organoid maintenance (9-35DD)

On 9DD, medium was removed from the device, and the device was dried using a Kimwipe (Kimberly-Clark). The device was placed on a clean cold pack for Matrigel embedment. Cold 66% Matrigel (100μL of 100% Matrigel with 50μL of N2+B27+Dorsomorphin medium) was injected into one of the side inlet holes uncovered by the membrane. An edge of a Kimwipe was inserted into the other inlet hole to draw the Matrigel across the chamber by capillary force. The chamber was successfully filled with the 66% Matrigel once the edge of the Kimwipe appeared pink. Any remaining 66% Matrigel was placed dropwise over each of the nine medial holes. The device was then placed back into the cell incubator for 25 minutes to allow the Matrigel to solidify in the chamber. After these 25 minutes, the device was filled with 3ml of N2+B27+Dorsomorphin medium. Medium was exchanged every other day until 14DD. On 14DD, medium was changed to N2+B27 medium and continued to be exchanged every other day until 35DD.

Confocal microscopy and analysis of microfabricated devices

Confocal imaging was conducted on 21DD, 28DD, and 35DD using a Nikon X1 Yokogawa spinning disk confocal and a Tokai Hit incubated stage to maintain device incubation. 20X and 40X water objectives were used with a 0.17 correction collar. The 20X objective was used to acquire images of the entire organoid using parameters of large area with a 15% overlap; z-stack defined with top and bottom coordinates of the organoid of ~100μm thick with z-steps of 5μm. The 40X objective was used to acquire images of single rosettes using a z-stack defined by top and bottom

coordinates through the lumen of the rosette with z-steps of 0.5μm. Low exposure times of ≤ 70ms and low laser power ≤ 8% were maintained to minimize bleaching and phototoxicity. The 40X objective was used for time-lapse microscopy at 28DD and 35DD when the organoids were in close proximity to the coverslip. Images were acquired every five to ten minutes for a duration of 8-16 hours over an 8-10μm z-stack range using a 0.5μm step. Multipoints were selected and perfect focus was applied to help maintain z-position throughout the entirety of the acquisition. Image analysis was completed using Nikon Elements Advanced Research software package.

Image processing and data analysis

Specimens were examined and the images acquired using a ZEISS Axio Scan.Z1 (Carl Zeiss S.p.A, Milano, Italy), E-800 Nikon microscope (Nikon, Melville, NY, USA) connected to a colour CCD Camera, and Eclipse 90i confocal microscope (Nikon, Melville, NY, USA). Adobe Photoshop 6.0 (Adobe Systems, San Jose, CA) was used to adjust image contrast. Quantitative evaluations were performed followed by different image analysis software: Microscope Software ZEN 3.0 lite (Carl Zeiss S.p.A, Milano, Italy), Neurolucida (MicroBrightfield, Colchester, VT) and ImageJ (Research Service Branch, National Institutes of Health, Bethesda, MD; available at http://rsb.info.nih.gov/ij/) analyses. Measurements were derived from at least three sections per mouse brain. Three to six animals were analyzed per each animal group, and values were averaged for each animal. The quantification for γH2AX density (cell number/mm^2), was done by selecting 9 fields for each genotype. For the neuroanatomical analysis, only the vermis slices were considered. For the cerebellar area and perimeter, the EGL and IGL area, slides colored in toluidine blue were used. For requiring quantifications, the section of the total cerebellum of three different sectors of the vermis–an anterior sector (lobules I - V), a central sector (lobules VI - VIII) and a posterior sector (lobules IX - X) were analyzed. Total area, EGL and IGL areas were calculated for each analyzed region. The anatomical analysis was performed using the 'Analyze Interactive' tool of Microscope Software ZEN 3.0 lite. The slices immuno-stained for Calbindin were analyzed for Purkinje Cell Linear Density quantification, using E-800 Nikon microscope with 20x objective. These quantifications were performed using MBF Bioscience's Neurolucida live: the Purkinje cell layer length was measured by drawing a freehand line through the center of the cell

bodies of cells in the layer, and the number of Purkinje cells (i.e., cell bodies) were quantified counting the Calbindin+ cells visible in each section. The number of Purkinje cells divided by the Purkinje cell layer length represents Purkinje cell linear density (i.e., Purkinje cells per mm of Purkinje cell layer). The quantification for cleaved caspase-3 was performed using Microscope Software ZEN 3.0 lite, and MBF Bioscience's Neurolucida live was used to quantify γH2AX. Organoid sections stained for CIT, MKLP1, Aurora B, cCASP3, KI67 and γH2AX were acquired using Nikon X1 Yokogawa spinning disk confocal or Nikon N-SIM + A1R inverted microscope. The quantifications were performed using ImageJ.
