# Supplementary figures and images for: Modeling primary microcephaly with human brain organoids reveals fundamental roles of CIT kinase activity
Source: J Clin Invest. 2024 Nov 1;134(21):e175435. doi: 10.1172/JCI175435 (PMC11527453; doi:10.1172/JCI175435)

**Full unedited gel for Figure 2**

**E**

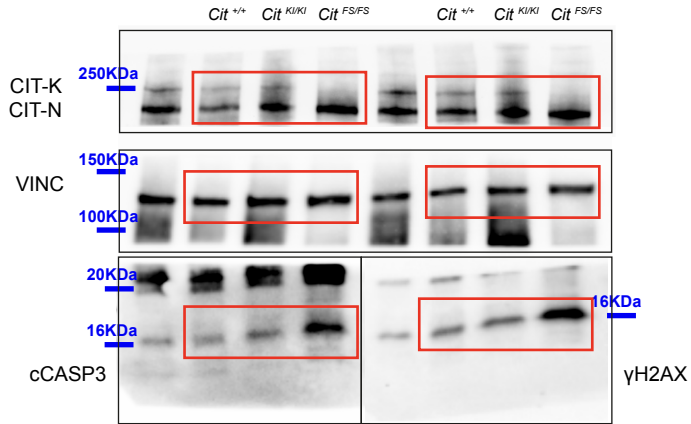

**Full unedited gel for Figure 3**

**C**

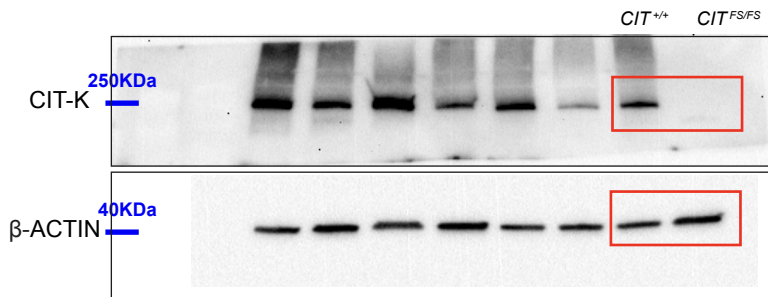

**D**

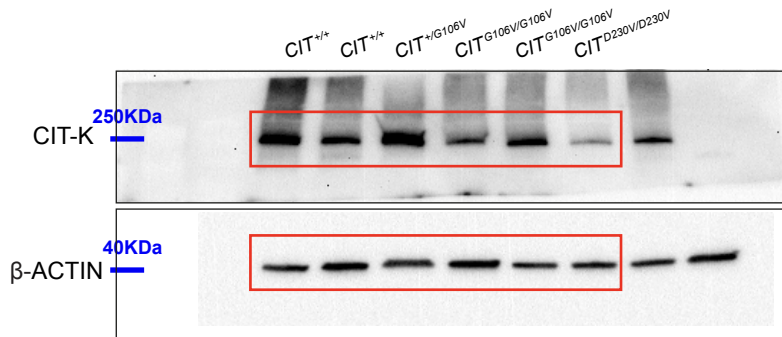

# Full unedited gel for Figure S1

**B**

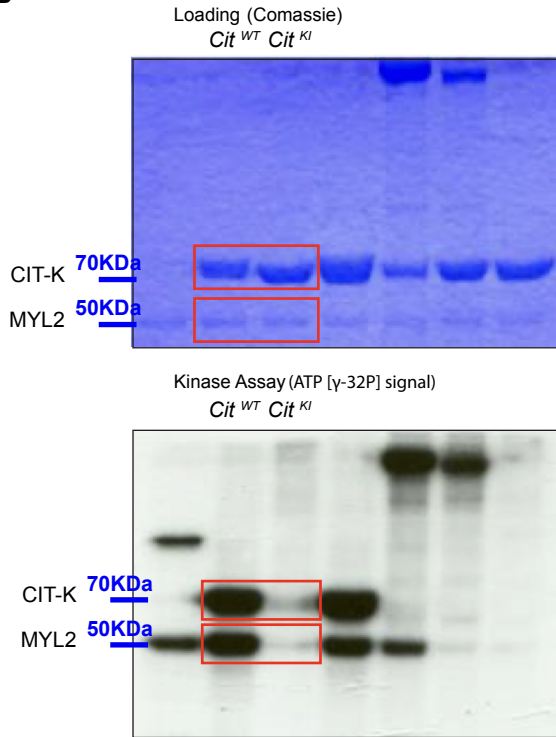

**E**

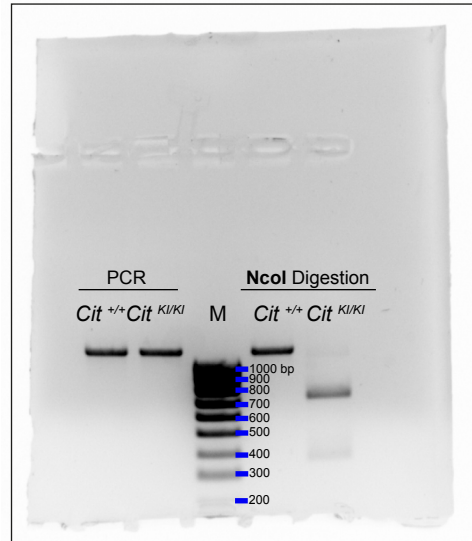

**F**

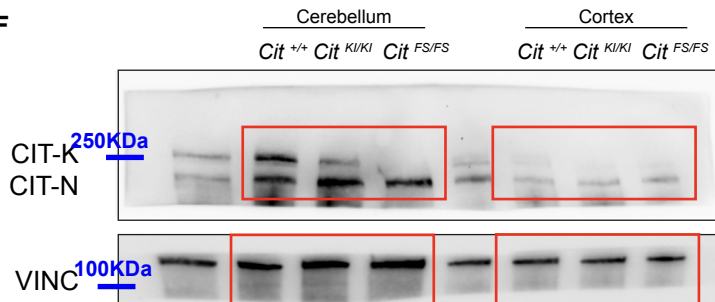

Supplement: Unedited blot and gel images [file jci-134-175435-s120.pdf]
